# Gene Editing of Hematopoietic Stem Cells: Hopes and Hurdles Toward Clinical Translation

**DOI:** 10.3389/fgeed.2021.618378

**Published:** 2021-03-31

**Authors:** Samuele Ferrari, Valentina Vavassori, Daniele Canarutto, Aurelien Jacob, Maria Carmina Castiello, Attya Omer Javed, Pietro Genovese

**Affiliations:** ^1^San Raffaele Telethon Institute for Gene Therapy (SR-Tiget), Istituto di Ricovero e Cura a Carattere Scientifico San Raffaele Scientific Institute, Milan, Italy; ^2^PhD course in Molecular Medicine, Vita-Salute San Raffele University, Milan, Italy; ^3^Pediatric Immunohematology and Bone Marrow Transplantation Unit, Istituto di Ricovero e Cura a Carattere Scientifico Ospedale San Raffaele, Milan, Italy; ^4^PhD Program in Translational and Molecular Medicine (DIMET), Milano-Bicocca University, Monza, Italy; ^5^Institute of Genetic and Biomedical Research Milan Unit, National Research Council, Milan, Italy; ^6^Division of Hematology/Oncology, Boston Children's Hospital, Boston, MA, United States; ^7^Department of Pediatric Oncology, Dana-Farber Cancer Institute, Boston, MA, United States; ^8^Harvard Stem Cell Institute, Cambridge, MA, United States; ^9^Department of Pediatrics, Harvard Medical School, Boston, MA, United States

**Keywords:** gene editing, hematopoietic stem cell, CRISPR/Cas, gene therapy, hematological diseases

## Abstract

In the field of hematology, gene therapies based on integrating vectors have reached outstanding results for a number of human diseases. With the advent of novel programmable nucleases, such as CRISPR/Cas9, it has been possible to expand the applications of gene therapy beyond semi-random gene addition to site-specific modification of the genome, holding the promise for safer genetic manipulation. Here we review the state of the art of *ex vivo* gene editing with programmable nucleases in human hematopoietic stem and progenitor cells (HSPCs). We highlight the potential advantages and the current challenges toward safe and effective clinical translation of gene editing for the treatment of hematological diseases.

## Introduction

Gene therapy aims to treat human diseases by modifying the cell genome e.g., by replacing a defective gene or providing a novel cellular function. In most cases, gene therapy exploits the knowledge on viral biology to generate recombinant vectors able to carry and transfer an exogenous coding cassette into patients' cells. The remarkable progresses in collection and *in vitro* manipulation of HSPCs have enabled the development of *ex vivo* gene therapy strategies, which confine the manipulation to a defined cell subset, thus diminishing the risk of off-target effects and bystander toxicity spillover. *Ex vivo* gene therapy based on semi-randomly integrating retro- (RV) or lenti-viral (LV) vectors has demonstrated an outstanding potential for the treatment of several inherited and acquired hematological diseases (Ghosh et al., 2015; Naldini, 2019). To this goal, autologous HSPCs are harvested, transduced *in vitro* by viral vectors and ultimately infused into the patient. A conditioning regimen is usually administered prior to infusion to deplete host cells and maximize engraftment of the engineered product (Bernardo and Aiuti, 2016).

The discovery and repurposing of programmable molecules, such as nucleases, base editors and prime editors have opened the door to targeted genome editing, i.e., site-specific nucleotide(s) deletion, insertion and substitution, or integration of a therapeutic transgene cassette at a pre-determined genomic locus (Doudna, 2020). These new technologies may be exploited to deliver a wide spectrum of genetic manipulations, with potential applications for several hematological diseases. Indeed, targeted genome editing by programmable nucleases has already entered the clinic and is currently being tested with encouraging results (Xu et al., 2019; Frangoul et al., 2021). While blossoming, gene editing is still in its infancy, and both knowledge and technological gaps await to be filled to broaden its clinical applicability. Furthermore, safety and efficacy, both in the short and long term, are still unknown.

In this Review, we highlight the therapeutic potential and the current challenges toward clinical translation of targeted genome editing by programmable nucleases in human HSPCs for the treatment of blood diseases.

## Programmable Nucleases for Targeted Genome Editing

Programmable nucleases are chimeric molecules composed by (i) a protein- or an RNA-based DNA binding structure, which dictates nuclease specificity, and (ii) an effector domain with catalytic nuclease activity, which induces a DNA double strand break (DSB) nearby or within the binding site. Zinc Finger Nucleases (ZFNs), Transcription Activator-Like Effector Nucleases (TALENs), and Clustered Regularly Interspaced Short Palindromic Repeats (CRISPR)/Cas systems are the most exploited nuclease platforms for targeted genome editing (Carroll, 2014).

ZFNs are composed by an array of three to six zinc-finger (ZF) DNA binding domains, linked by a flexible peptide linker to a non-specific FokI cleavage domain. Each ZF domain is composed by 30 amino acids and recognizes nucleotide triplets in the major groove of the DNA double helix; in total each ZFN recognizes 9–18 nucleotides (Gaj et al., 2013). Sequence and structure of the aforementioned flexible peptide linker is fundamental to achieve efficient cleavage and targeting specificity (Handel and Cathomen, 2011). Mechanistically, a pair of ZFN monomers must bind the DNA, typically in a head-to-head configuration, by associating with DNA strands of opposite polarity and leaving a 5–7 bp gap. This leads to dimerization of the two FokI domains that catalyze the DNA DSB (Urnov et al., 2010).

TALENs consist of a DNA-binding domain composed by modular TALE repeats, fused with a FokI nuclease domain. Each TALE repeat is composed by 33–35 amino acids and recognizes a single nucleotide; specificity is determined by two hypervariable residues, known as Repeated Variable Diresidues (RVDs) (Gaj et al., 2013). Indeed, TALE repeats can be assembled together in a rather straightforward way to pair the desired DNA sequence, nucleotide by nucleotide. As for ZFNs, a pair of TALEN monomers is necessary to introduce a DSB.

Finally, CRISPR/Cas is an RNA-based DNA targeting-system found in bacteria as an acquired immune system against transmissible genetic elements, such as viruses and plasmids (Barrangou et al., 2007; Brouns et al., 2008; Garneau et al., 2010). *Streptococcus pyogenes* (Sp) Cas9 protein (SpCas9) (Nozawa et al., 2011), which belongs to type II family of CRISPR/Cas systems, is the most widely used platform for CRISPR-based targeted genome editing. Mechanistically, the CRISPR/Cas9 system is composed by a single-stranded guide RNA (sgRNA) and the Cas9 endonuclease, which is the enzyme required to mediate target DNA cleavage. The sgRNA contains a unique 20 base-pair sequence which complements the target DNA site, and can be easily customized to bind the desired genomic sequence by Watson-Crick base-pairing (Jinek et al., 2012). The presence of a protospacer adjacent motif (PAM), immediately downstream the target DNA site, is necessary to efficiently bind and cut the DNA, e.g. 5′-NGG-3′ for SpCas9, although some cleavage activity has also been observed with the 5′-NAG-3′ motif (Hsu et al., 2013; Sternberg et al., 2014).

All these platforms have intrinsic advantages and disadvantages (Gaj et al., 2013). TALENs can be easily assembled in arbitrarily large arrays to bind the sequence of interest, but their intrinsic repetitiveness and large size impair efficient cloning and limit delivery by viral vectors. ZFNs are relatively smaller in size and easier to clone, but difficult to design and optimize, due to the lack of a stringent recognition code and the interdependence of each module with the surrounding ones. Both tools have a limited range of targetable DNA sequences as ZFNs prefer G-rich sequences (Isalan, 2012), while TALENs typically bind low G content sites strictly beginning with a T base (Bogdanove and Voytas, 2011). Instead, the CRISPR/Cas9 system is more flexible, and targeting is usually easier and faster, as it suffices to design and synthetize a sgRNA complementary to the sequence(s) of interest. Multiple sequences may be targeted simultaneously, and no protein optimization is required. Because of its features, the popularity of CRISPR/Cas technology rapidly surpassed that of ZFNs and TALENs.

Still, CRISPR/Cas9 is not free from limitations, the main being the distribution of PAM sequences, that constrains the set of targetable sequences. Indeed, huge efforts have been made to expand the repertoire of potential targets. Cas9 homologs (e.g., Cas12a/Cpf1) (Zetsche et al., 2015a) and Cas9 proteins requiring different PAM sequences have been identified in other bacteria species (Ran et al., 2015; Xu et al., 2015; Lee et al., 2016; Müller et al., 2016); Cas9 variants with relaxed PAM preferences (Cas-NG and xCas) (Hu et al., 2018; Nishimasu et al., 2018) or unconventional PAM profiles (SpCas9-VQR, VRQR and VRER) (Kleinstiver et al., 2015, 2016) have been developed by directed evolution or structure-guided engineering. Recently, a SpCas9 variant (SpRY), requiring a 5′-NRN-3' PAM, has been generated to edit previously inaccessible genetic sites, significantly overcoming most PAM-related limitations (Walton et al., 2020). To date, little data has been generated in primary blood cell types with the aforementioned tools (Wang et al., 2017; Xiao et al., 2019), which would thus require further validation to be employed for hematological diseases.

## Therapeutic Opportunities for Targeted Genome Editing in HSPCs

**Figure 1:**
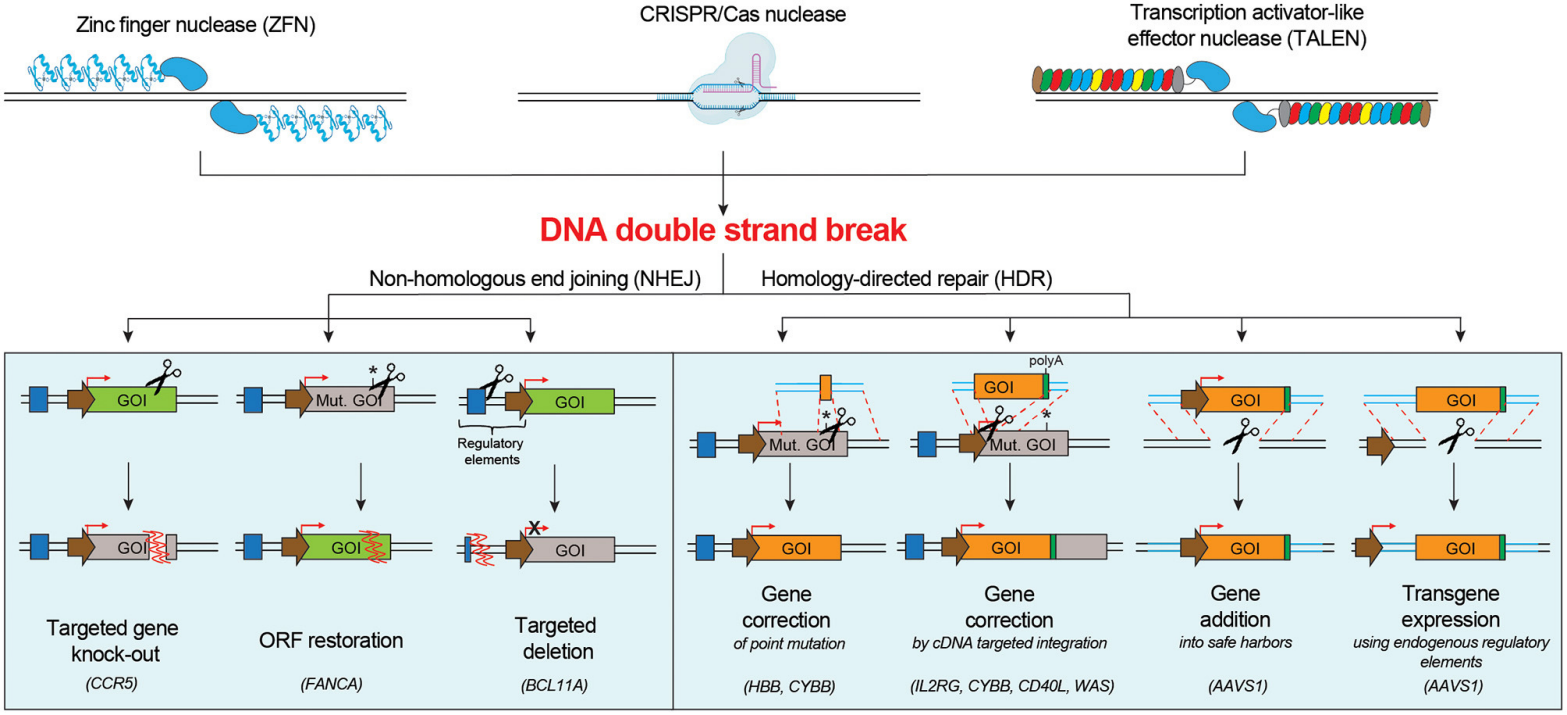
Schematic of the main DNA double strand break repair mechanisms in human cells and their possible applications for targeted genome editing. NHEJ pathway engagement may be exploited for knocking-out a gene, correcting a gene by restoring its open reading frame (ORF), inserting a targeted deletion. HDR pathway engagement may be exploited for gene correction of point or multiple mutations, gene addition in a safe harbor locus or targeted transgene expression using endogenous regulatory elements. Mut. GOI, Mutated Gene Of Interest.

Induction of one or multiple DNA DSB(s) by programmable nucleases triggers the DNA damage response (DDR), which mediates DNA repair and ultimately defines cell fate. DNA DSB repair mainly occurs by the non-homologous end joining (NHEJ) pathway or the homology-directed repair (HDR) pathway (Chapman et al., 2012), although alternative pathways have also been described (Yeh et al., 2019). The NHEJ machinery stitches the broken DNA ends in an error prone way, often by deleting or inserting random bases (indels). Instead, the HDR pathway exploits a homologous DNA template, like the sister chromatid, to faithfully repair the DNA breaks. While NHEJ is active throughout the cell cycle, HDR is confined to S/G2 phases (Branzei and Foiani, 2008; Heyer et al., 2010). Both NHEJ- and HDR-mediated repair of nuclease-induced DNA DSBs have been explored for therapeutic purposes in HSPCs (Figure 1).

NHEJ-based genome editing finds the following applications: (**i) targeted gene knock-out**: NHEJ-mediated indels directed to a coding sequence may result in frameshift mutations and generation of premature stop codons, which can render the targeted gene non-functional. This strategy can be used to silence a pathogenic gene or induce resistance against a pathogen by knocking out genes that facilitate infections, e.g., the disruption of the *CCR5* open reading frame in hematopoietic cells to confer resistance to HIV infection (Wang and Cannon, 2016; Xu et al., 2019); (**ii) restoration of the correct reading frame**: NHEJ-mediated indels can be exploited to restore the normal reading frame of a gene and thus correct frame-shift mutations. This strategy may be suitable to correct some Fanconi Anemia disease-causing mutations on *FANCA* gene in HSPCs (Román-Rodríguez et al., 2019); (**iii) introduction of a targeted deletion**: programmable nucleases can be used to create two DBSs flanking a region of interest, which is excised and deleted as the gap is repaired by NHEJ (Lee et al., 2010). This strategy can be exploited to remove one or more pathogenic exons, to cut dominant triplet expansion or to delete a regulatory region that alters protein expression. Deletion of the erythroid specific enhancer of *BCL11A* (Bauer et al., 2013), a transcriptional repressor that inhibits fetal hemoglobin (Hb-F) expression, can enhance the levels of Hb-F, resulting in phenotype alleviation of sickle cell disease (SCD) and β-thalassemia.

HDR-mediated genome editing requires the supply of a DNA donor template, harboring homologous sequences with the nuclease target site, and may be exploited for the following applications: **(i) targeted correction of point mutations**: delivery of a nuclease that cleaves close to the mutation site and of a donor template containing the wild-type sequence (Urnov et al., 2005) can be exploited to correct single nucleotide mutations. This approach may be suitable for SCD, which is caused by a single amino acid substitution (Glu to Val) in the sixth position of the *HBB* gene (Dever et al., 2016; DeWitt et al., 2016; Park et al., 2019; Pattabhi et al., 2019; Romero et al., 2019), and for X-linked chronic granulomatous disease (CGD), often caused by mutations in the *CYBB* gene (De Ravin et al., 2017, 2021); **(ii)** ***in situ* gene correction by targeted insertion of a cDNA**: many monogenetic diseases are not caused by a recurrent single nucleotide mutation, but rather different mutations affecting the same gene. Integration of a functional cDNA, spanning the mutation hotspots, in the intended region of the target gene (e.g., endogenous start codon, intronic region), can simultaneously bypass all downstream mutations (Voit et al., 2014). Proof-of-principle of this approach has been demonstrated for several hematological diseases, including X-linked severe combined immunodeficiency (SCID-X1) (Schiroli et al., 2017; Pavel-Dinu et al., 2019), CGD (Sweeney et al., 2017), Hyper-IgM 1 syndrome (Hubbard et al., 2016; Kuo et al., 2018; Vavassori et al., 2021) and Wiskott-Aldrich syndrome (WAS) (Rai et al., 2020); **(iii) targeted gene addition into a safe harbor locus**: integration of a therapeutic cassette in a specific region of the genome might represent a valuable strategy when constitutive overexpression of the transgene is required in order to obtain a therapeutic effect (Moehle et al., 2007). The best locations for gene addition in the genome are genomic safe harbors, categorized as genomic locations that are tolerant to homozygous gene inactivation, support robust transgene expression, and tolerate integration of the transgene and its regulatory elements without causing any adverse effects, such as malignant transformation or altered cellular function (Sadelain et al., 2012). One representative application is the targeted integration of corrective *gp91phox* transgene in *AAVS1* locus for treating CGD (De Ravin et al., 2016); **(iv) transgene expression using endogenous regulatory elements**: control of transgene expression by endogenous regulatory elements can provide high, robust and cell specific expression of proteins. Examples are α-L-iduronidase (Hurler syndrome, OMIM #607014), α-galactosidase (Fabry disease, OMIM #301500), lysosomal acid lipase (Wolman disease, OMIM #278000), and factor IX (Hemophilia B, OMIM #306900), that under the transcriptional control of the endogenous α-globin promoter resulted in erythroid-specific expression (Pavani et al., 2020).

## Challenges And Advances Toward Clinical Application Of HSPC Gene Editing

Preliminary results encourage clinical translation of HSPC gene editing for blood disorders. However, a number of issues must be addressed, ranging from sourcing and culturing of the cells, delivery of the nucleases and the corrective template, nuclease activity, and efficiency of gene correction, particularly in the long-term repopulating HSC fraction. Depending on the disease setting, each of these aspects must be accounted for comprehensive risk/benefit evaluation of the therapeutic strategy. Several studies in the last years were focused on the optimization of the editing protocol and the development of novel tools and strategies to maximize editing efficiency and specificity for its safe and successful empowerment.

### Tailoring *ex vivo* Cell Culture Conditions

**Figure 2:**
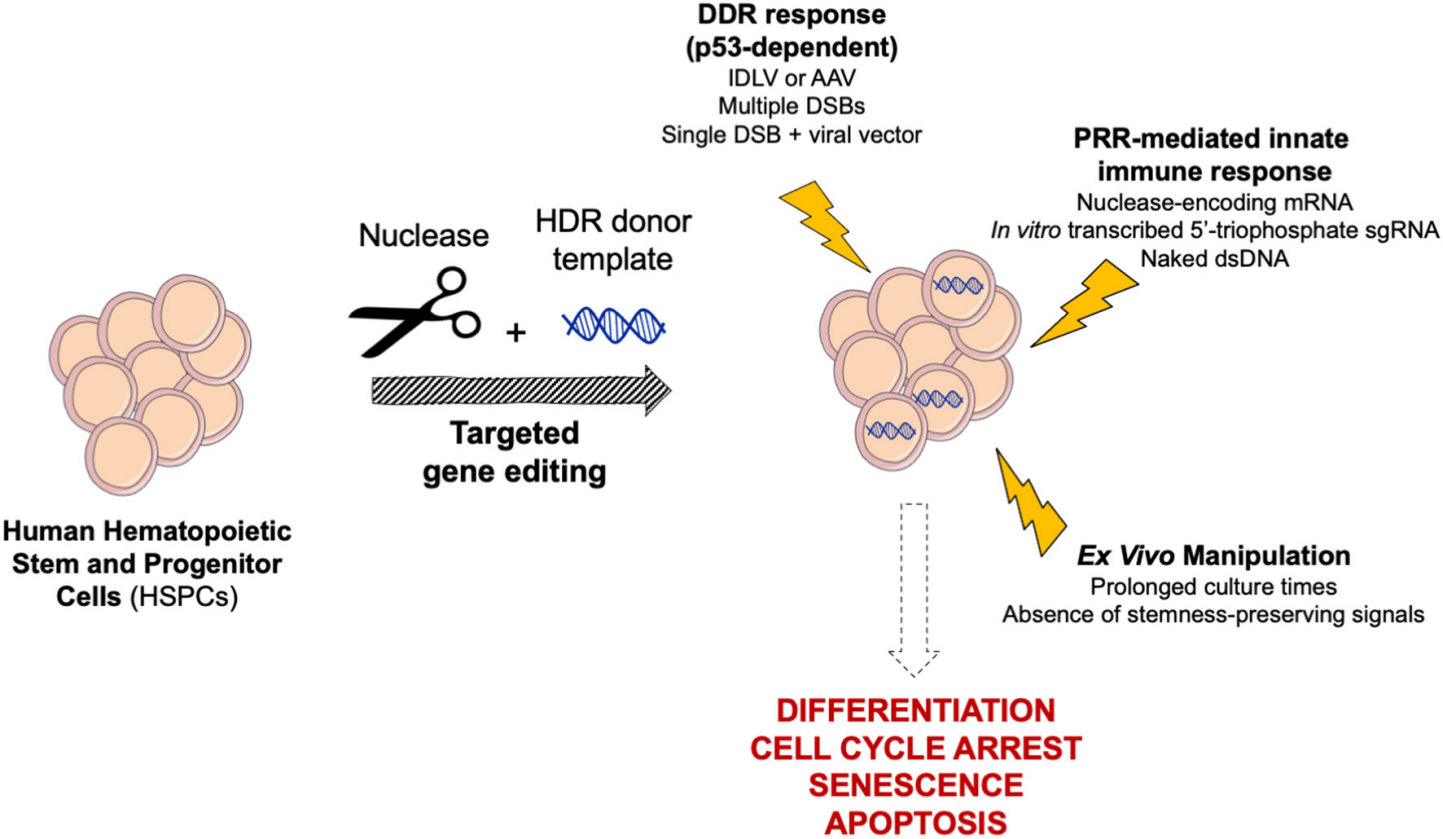
Schematic of cellular responses triggered by targeted genome editing in human HSPCs. Gene editing reagents, procedure and *ex vivo* manipulation may trigger complex cellular responses in HSPCs, ultimately leading to differentiation, cell cycle arrest, senescence, and apoptosis. DDR, DNA Damage Response; PRR, Pattern Recognition Receptor.

Cell culture protocols must strike a balance between permissiveness to editing manipulation, cell expansion and maintenance of the stemness potential (Figure 2). Fine-tuning of culture conditions and editing timing have been pursued to promote HSPC cell cycle progression and activation, and to achieve sustained editing, while preserving long-term persistence of engineered cells. Indeed, HDR-mediated gene editing is constrained in slowly cycling and quiescent primitive HSCs. Generally, the expression level of the DNA repair machinery correlates with the cell proliferation activity and stemness; therefore, long-term repopulating HSCs show lower permissiveness to HDR than committed progenitors (Beerman et al., 2014; Biechonski et al., 2018; Schiroli et al., 2019). *Ex vivo* culture of HSPCs for 48 or 72 h before editing, in presence of cytokine mixtures containing at least SCF, FLT-3L, and TPO (Walasek et al., 2012), pushes repopulating cells to exit from quiescence and transit through S/G_2_ phases, thus increasing HDR efficiency (Genovese et al., 2014; Zonari et al., 2017; Bak et al., 2018). However, prolonged culture times lead to cell differentiation and multipotency loss. Supplementation of the culture medium with stemness preserving compounds, such as Stem Regenin-1 (Boitano et al., 2010), UM171 (Fares et al., 2014) and 16,16-dimethyl prostaglandin E2 (dmPGE_2_) (Hoggatt et al., 2009), helps to maintain the long-term multilineage repopulation capacity of human edited HSPCs transplanted in immunodeficient mouse models, partially overcoming the drawbacks of prolonged culture (Charlesworth et al., 2018; Ferrari et al., 2020).

### Delivery Vehicles for Programmable Nucleases and DNA Template for HDR-Mediated Editing

Several platforms have been tested to deliver the programmable nucleases and the HDR template in hematopoietic cells with the ultimate goals of maximizing editing efficiency and minimizing treatment toxicity. The proof-of-concept for *in vitro* HDR-mediated integration has been made by delivering both the nuclease and the donor cassette with viral vectors in human HSPCs (Lombardo et al., 2007). Later electroporation became the method of choice to efficiently deliver programmable nucleases in *ex vivo* cultured HSPCs (Genovese et al., 2014). *In vitro* transcribed mRNA encoding for the nucleases (Genovese et al., 2014; Wang et al., 2015; Schiroli et al., 2017) or ribonucleoprotein (RNP) assembled with recombinant Cas protein and sgRNA (Hendel et al., 2015; Dever et al., 2016) have become the gold standard to achieve a high but transient nuclease activity in HSPCs and other target cells (Hubbard et al., 2016; Eyquem et al., 2017). Transduction with viral vectors as integrase-defective LVs (IDLVs) or adeno-associated vectors serotype 6 (AAV6) (Genovese et al., 2014; Wang et al., 2015; Dever et al., 2016; Schiroli et al., 2017; Kuo et al., 2018; Pavel-Dinu et al., 2019; Rai et al., 2020), as well as the electroporation of single-stranded phosphorothioate-modified oligodeoxynucleotides (ssODNs) (DeWitt et al., 2016; De Ravin et al., 2017, 2021; Park et al., 2019; Pattabhi et al., 2019; Romero et al., 2019), are the vehicles currently preferred to deliver the DNA template for HDR in HSPCs. Overall, these platforms offer a broad spectrum of cargo capacities and may be suitable for different editing strategies. Short ssODN are limited in length and may be applied for *in situ* gene correction of small disease-causing mutations. Conversely, AAV6 and IDLV welcome larger payloads (approximately up to 4.7 and 8 kb, respectively), suitable for targeted integration of long therapeutic cassettes. Instead, adenoviral vectors and other non-viral vehicles, such as plasmids and double-stranded DNA templates, found limited applications in primary hematopoietic cells due to poor efficiency and tolerability, albeit with some exceptions (Roth et al., 2018).

### Maximizing HDR Editing Efficiency

The absolute and relative numbers of cells that need to be edited depend on the disease and on the therapeutic strategy. For instance, as the absence of IL2RG is lethal for developing lymphocytes, the strong selective advantage of functional T cell progenitors over affected ones may compensate for relatively low editing efficiencies, and <10% of functional HSPCs are predicted to be sufficient to rescue the SCID-X1 phenotype (Schiroli et al., 2017). Conversely, the minimal proportion of edited cells must be substantially higher to fully rescue the pathological features of patients affected by other blood disorders, such as hemoglobinopathies or Hyper-IgM1 (Abraham et al., 2017; Marktel et al., 2019; Vavassori et al., 2021; Wilkinson et al., 2021). Indeed, suboptimal HDR editing efficiency remains a major constrain for broader application of this technology, as opposed to the high efficiency of NHEJ-mediated editing (Humbert et al., 2019; Frangoul et al., 2021).

In pioneering gene editing studies on human HSPCs, IDLV transduction combined with ZFNs mRNA electroporation led to 5–10% HDR editing in the bulk CD34+ population and 2–5% in the primitive CD34+CD133+CD90+ HSPC fraction, which entails cells with long-term engraftment capacity in immunodeficient mice (Genovese et al., 2014). Instead, switching to AAV6 vectors for HDR DNA template delivery has allowed increasing up to 5-fold the HDR editing efficiency in primitive HSPCs compared to the IDLV-based protocol, regardless of the nuclease platforms employed (Wang et al., 2015; Dever et al., 2016; Schiroli et al., 2017; Kuo et al., 2018; Pavel-Dinu et al., 2019; Rai et al., 2020). Of note, IDLV transduction in presence of cyclosporin H enhanced HDR efficiency up to 15–20% in the long-term progeny of human engrafting HSPCs by relieving interferon-induced transmembrane protein 3 (IFTM3)-mediated entry restriction (Petrillo et al., 2018; Soldi et al., 2020), thus suggesting that the total DNA load into the nucleus of transduced cells is still a limiting step for HDR engagement. Nevertheless, other molecular mechanisms enhancing HDR efficiency with AAV6 still remain partially elusive. Recruitment of HDR factors by AAV inverted terminal repeats (Hirsch, 2015) and engagement of alternative pathways exploiting single-stranded templates for DNA DSB repair may contribute to the enhancement of HDR editing (Yeh et al., 2019). Of note, ssODNs allow for gene correction efficiencies similar to those obtained with AAV6 in primitive HSPCs long term after xenotransplantation (De Ravin et al., 2017; Pattabhi et al., 2019; Romero et al., 2019). ssODNs likely engage DNA DSB repair mechanisms distinct from those of IDLV and possibly AAV6, preferring the single-stranded template repair (SSTR) pathway rather than the conventional HDR (Richardson et al., 2018).

Despite these substantial steps forward, HDR editing efficiency is still limited for some applications. Several strategies were proposed to enhance HDR efficiency in mammalian cells by transiently manipulating the DNA repair pathways or the cell cycle status. NHEJ inhibition by small molecules or proteins, tethering of HDR-promoting factors to Cas9 nuclease, or S/G2 cell synchronization, favored HDR engagement upon nuclease-induced DNA DSB in cell lines and pluripotent cells (Chu et al., 2015; Maruyama et al., 2015; Gutschner et al., 2016; Charpentier et al., 2018; Jayavaradhan et al., 2019). However, the efficacy of these approaches in long-term repopulating HSCs has been limited (Kuo et al., 2018; De Ravin et al., 2021) or unproven. Recently, promoting cell cycle progression, either by maintaining low cell concentration during *ex vivo* manipulation (Charlesworth et al., 2018) or with cell-cycle modulators (Ferrari et al., 2020; Shin et al., 2020), has been reported as the most efficient strategy to enhance HDR editing in human long-term repopulating HSCs.

Enrichment for cells undergoing the intended genome modification may be an alternative (or even complementary) strategy to increase the proportion, but not the number, of edited cells in the graft and reduce the competition with the unedited counterpart. Gene correction may be amenable to sort for edited cells by exploiting endogenous markers expressed on the cellular membrane. As a paradigmatic example, selection of *HBB*-edited HSPCs was achieved by embedding a reporter cassette in the HDR template, reaching up to 90% HDR-edited cells in the long-term graft (Dever et al., 2016). In another study, simultaneous editing of the locus of interest and of an unrelated gene providing drug-resistance to chemical compounds allowed to efficiently enrich for human edited HSPCs in marker-free settings (Agudelo et al., 2017).

### Tolerability of the Gene Editing Procedure

To counter the constant threat of DNA damaging agents, cells have evolved a panel of repair mechanisms, as well as senescence and programmed cell death pathways. Indeed, both the DSB and the delivery of an exogenous donor template that are instrumental to gene editing may trigger complex cellular responses potentially leading to harmful outcomes (Figure 2). However, the consequences of gene editing on cell fitness, as well as NHEJ/HDR proficiency, may vary across different cell types and strongly depend on cell biology. Likely due to their fundamental role in blood homeostasis, human HSPCs are evolutionarily more sensitive than cell lines and other cell types to extensive manipulation; therefore, the gene editing procedures have to be substantially tailored to maximize tolerability.

As a first line of host defense, human immune cells exhibit pattern recognition receptors (PRRs), which sense pathogen-associated molecular patterns (PAMPs), such as exogenous nucleic acids, and promote the release of type I interferons (IFNs) and other cytokines (Piras and Kajaste-Rudnitski, 2020). Activation of PRRs in HSPCs and overexpression of IFN-stimulated genes (ISGs) can induce a variety of outcomes, including exit from quiescence, differentiation and apoptosis (Essers et al., 2009; Sato et al., 2009; Liu et al., 2012). *In vitro* transcribed 5′-triphosphate sgRNAs and mRNAs encoding for nucleases may strongly activate ISGs via PRRs, decreasing cell viability and clonogenic potential (Mu et al., 2019). Dampening of these responses has been obtained by switching to chemically synthetized sgRNAs or high-pressure liquid chromatography (HPLC)-purified mRNAs incorporating base analogs (Hendel et al., 2015; Schiroli et al., 2017). Furthermore, electroporation of CRISPR/Cas9 machinery as RNP, rather than mRNA, is reported to be stealthier in human HSPCs (Cromer et al., 2018). Of note, IFN induction may also affect concomitant viral transduction, thus constraining HDR template delivery for some vectors (Petrillo et al., 2018). Moreover, secondary structures or nucleic acid hybrids present in viral vector genomes may be recognized by the host and activate transient cellular responses (Piras and Kajaste-Rudnitski, 2020).

Cell sensors are triggered not only by the presence of exogenous proteins and nucleic acid, but also by the DNA damage evoked by their action. Nuclease toxicity mediated by p53 was in fact observed in: (i) induced pluripotent stem cells (iPSCs) (Ihry et al., 2018) and cell lines (Haapaniemi et al., 2018; Enache et al., 2020), leading to apoptosis or cell cycle arrest; (ii) HSPCs with a remarkable impact on clonogenic capacity (Schiroli et al., 2019). Accordingly, multiple DSBs resulted in a higher p53-dependent DDR in HSPCs, up to the establishment of pro-inflammatory transcriptional programs, with corresponding higher impact on the clonogenic potential (Schiroli et al., 2019).

Both AAV6- and IDLV-mediated template delivery are sensed by HSPCs (Piras et al., 2017; Schiroli et al., 2019). In particular, AAV6 transduction per se triggers a robust p53 response, which cumulates with the one elicited from concomitant exposure to nucleases. These convergent inputs lead to substantial HSPC proliferation slowdown, shrinkage of the human graft size and oligoclonal reconstitution by edited cells upon xenotransplantation in immunodeficient mice (Ferrari et al., 2020). The molecular cascade leading to p53 activation has not been fully elucidated yet. Interestingly, however, transient p53 inhibition confined in the first 24 h of the editing process enhanced tolerability of the procedure and restored polyclonal composition of the human graft, preserving HSPC multilineage potential (Schiroli et al., 2019; Ferrari et al., 2020). Moreover, p53 inhibition may mitigate the theoretical risk of increasing the proportion of p53^−/−^ mutant clones with high oncogenic potential, which are typically rare in a HSPC population from healthy donors but could be more frequent in patients with specific genetic diseases, such as Fanconi anemia or Diamond-Blackfan anemia (Lipton and Ellis, 2009; Ceccaldi et al., 2011). While a transient p53 inhibition raises the theoretical concern of inappropriately rescuing cells with chromosomal aberrations and high mutational burden, no increase in the mutational load was reported by its incomplete and transitory inhibition (Garaycoechea et al., 2018; Schiroli et al., 2019). Moreover, even if some rare genotoxic event occurred, prompt restoration of the p53 pathway may be expected to counter-select cells that have acquired them before the occurrence of the subsequent hits necessary for oncogenic transformation (Di Micco et al., 2006; Bondar and Medzhitov, 2010).

Conversely, the use of ssODN instead of viral vectors as HDR template does not cumulatively elicit p53 activation and is well-tolerated by HSPCs, with no impact on their repopulation capacity (Pattabhi et al., 2019; Romero et al., 2019).

### Assessment of Editing Genotoxicity and Optimization of Nuclease Specificity

Specificity of programmable nucleases is defined as the ratio between on-target and off-target activity, i.e., the DNA DSB frequency at the intended target site and at unintended genomic loci. Although genome editing offers higher level of specificity than genetic engineering platforms based on semi-randomly integrating vectors, off-target generation of DNA DSBs could be a major source of genotoxicity. Nuclease off-target activity may have no biological consequences, or instead be cytotoxic, knock-out tumor suppressor genes, induce off-target incorporation of the donor DNA, or trigger chromosomal rearrangements. Its burden may vary depending on the nuclease platform, donor DNA and the targeted DNA sequence. Moreover, unintended on-target events, such as excision or insertion of arbitrary DNA fragments, have been reported upon gene editing in non-hematopoietic cell types (Kosicki et al., 2018; Hanlon et al., 2019; Nelson et al., 2019). The consequences of unintended on- or off-target events are expected to differ depending on the overall editing strategy and disease setting, and thus require case-by-case evaluation. Furthermore, off-target events may be presumed to be more tolerated in fully differentiated and short-lived cell types. Hence, careful assessment of nuclease specificity is mandatory when aiming to clinical translation of engineered HSPCs because these cells will have to support life-long hematopoiesis by performing several cycles of self-renewal and differentiation in the patient.

Given the hit and run nature of the programmable nucleases, comprehensive detection of off-target activity requires the development of innovative and specific tools. To this goal, a panel of *in silico* prediction algorithms (Haeussler et al., 2016; Labun et al., 2019) as well as *in vitro* (e.g., DIGENOME-seq, CIRCLE-seq) (Kim et al., 2015; Tsai et al., 2017) and *in cellulo* assays (e.g., IDLV trapping, GUIDE-seq) (Gabriel et al., 2011; Tsai et al., 2015) have been developed. All these methods show considerable sensitivity and specificity issues and none of them alone allows to comprehensively and precisely identify nuclease off-target sites, even because no gold standard exists. Indeed, bioinformatic tools and *in vitro* assays typically return a large number of putative off-target sites, but many of them do not overlap with those either found by *in cellulo* assays or validated by targeted next generation sequencing (NGS). The combination of more than one assay is generally advised in order to collect a broader panel of putative off-target sites that can be then validated by targeted NGS in the cell type of interest. On the other hand, it is debatable whether nuclease off-target sites revealed by *in silico* or *in vitro* assays but not confirmed by *in cellulo* assays or during the validation phase should be considered as false-positive events. The NGS detection limit (0.1–0.01%) is likely a major limitation toward comprehensive assessment of the off-target nuclease activity in view of clinical translation. For instance, up to 10^5^ cells in the drug product might have unmeasurable nuclease activity at an off-target site considering the dose of edited HSPCs commonly administered in gene therapy settings (from 10^7^ to 10^9^) (Gaspar et al., 2011; Sessa et al., 2016; Eichler et al., 2017; Thompson et al., 2018; Ferrua et al., 2019; Marktel et al., 2019; Esrick et al., 2021). Furthermore, specificity analyses usually do not take into account the wide spectrum of genomic polymorphism in the human population, thus likely dropping out potentially relevant individual- and population-specific off-target sites. *Ad hoc* assays to stringently and comprehensively assess the genotoxicity profile of programmable nucleases, on top and beyond the off-target events, are currently lacking and would be of relevance to address any safety concern at preclinical stage. Although no guidelines currently exist, previously validated unintended on- and off-target events should be strictly monitored after infusion in patients, similarly to longitudinal integration site analyses that are considered a standard in current gene addition clinical protocols (Aiuti et al., 2013; Thompson et al., 2018).

Off-target activity depends on: (i) the sequence homology between on- and off- target sites; (ii) the DNA affinity of the nuclease; (iii) the duration of exposure. To minimize off-target events, several strategies have been pursued, leveraging the last two aspects, as well as sgRNA screening to identify those predicted being more specific for targeting the intended region. Indeed, prolonged nuclease activity and high nuclease concentration decrease editing specificity (Hsu et al., 2013; Pattanayak et al., 2013; Kim et al., 2014; Shapiro et al., 2020), which instead can be improved by transient nuclease expression, e.g. via mRNA or RNP (Dever et al., 2016), or the use of split or inducible Cas9 mutants (Davis et al., 2015; Nihongaki et al., 2015; Zetsche et al., 2015b).

In the last years, extensive engineering improved CRISPR/Cas9 specificity and efficiency by modifying sgRNA and Cas9 architecture. The use of 5′-truncated sgRNAs (tru-gRNA) resulted in similar on-target activity as standard ones but several-fold lower off-target activity (Fu et al., 2014), likely reducing the interaction energy at the RNA–DNA heteroduplex level (Lim et al., 2016). The addition of two guanines at the 5′ end of the sgRNA also reduced off-target activity, albeit also decreasing on-target editing in some cases (Cho et al., 2014). Instead, chemical modifications (2′-O-methyl-3′phosphorothiorate or 2′-O-methyl-3′thiophosphonoacetate) of the three terminal nucleotides at the 5′ and 3′ ends improved the specificity profile and enhanced tolerability compared to unmodified sgRNA in hematopoietic cells (Hendel et al., 2015). As for what concerns Cas9, novel variants [eSpCas9(1.1) and SpCas9-HF1] with higher fidelity resulting from dampened interaction strength with the DNA (Kleinstiver et al., 2016; Slaymaker et al., 2016), have been identified by structure-guided mutagenesis. However, these variants showed lower on-target activity than wild-type SpCas9 in human HSPCs when delivered as RNP (DeWitt et al., 2016; Vakulskas et al., 2018). Recently, other highly specific SpCas9 variants, such as EvoCas9 (Casini et al., 2018), SniperCas9 (Lee et al., 2018) and HiFi-Cas9 (Vakulskas et al., 2018) were discovered by directed evolution approaches. The latter showed improved fidelity and high on-target editing over wild-type SpCas9 in human HSPCs. Similar approaches have been also pursued for other gene editing tools, including ZFNs and TALENs (Miller et al., 2007; Hubbard et al., 2015).

Although detailed analyses and considerations on nuclease specificity are imperative, the presence of some unwanted genomic events does not necessarily preclude gene editing from proceeding toward safe clinical applications. Precaution dictates that efforts should be made toward minimizing their incidence, but their actual consequences may depend on the specific context. An unintended genomic event may in theory contribute to cancer, contingently with its genomic location and its nature. However, since oncogenic transformation is multifaceted and multistep, the same mutations may or not give rise to tumors also depending on genetic background and the subsequent exposure to other genotoxic events. For reference, pathogenic mutations may remain silent for many years, and result in overt disease in a small fraction of individuals who endure another genomic “hit” (Greaves, 2018). Moreover, healthy individuals may harbor clonal hematopoiesis due to oncogenic mutations which can persist for years without evidence of pathogenicity despite increasing the risk of malignant transformation (Takahashi et al., 2017; Zink et al., 2017). On the contrary, in disease settings in which clonal expansion of the edited cells is already triggered by a strong selective advantage conferred by gene correction, the same genotoxic events might promote oncogenic transformation.

### Manufacturing for Therapeutic Gene Editing Protocol

**Figure 3:**
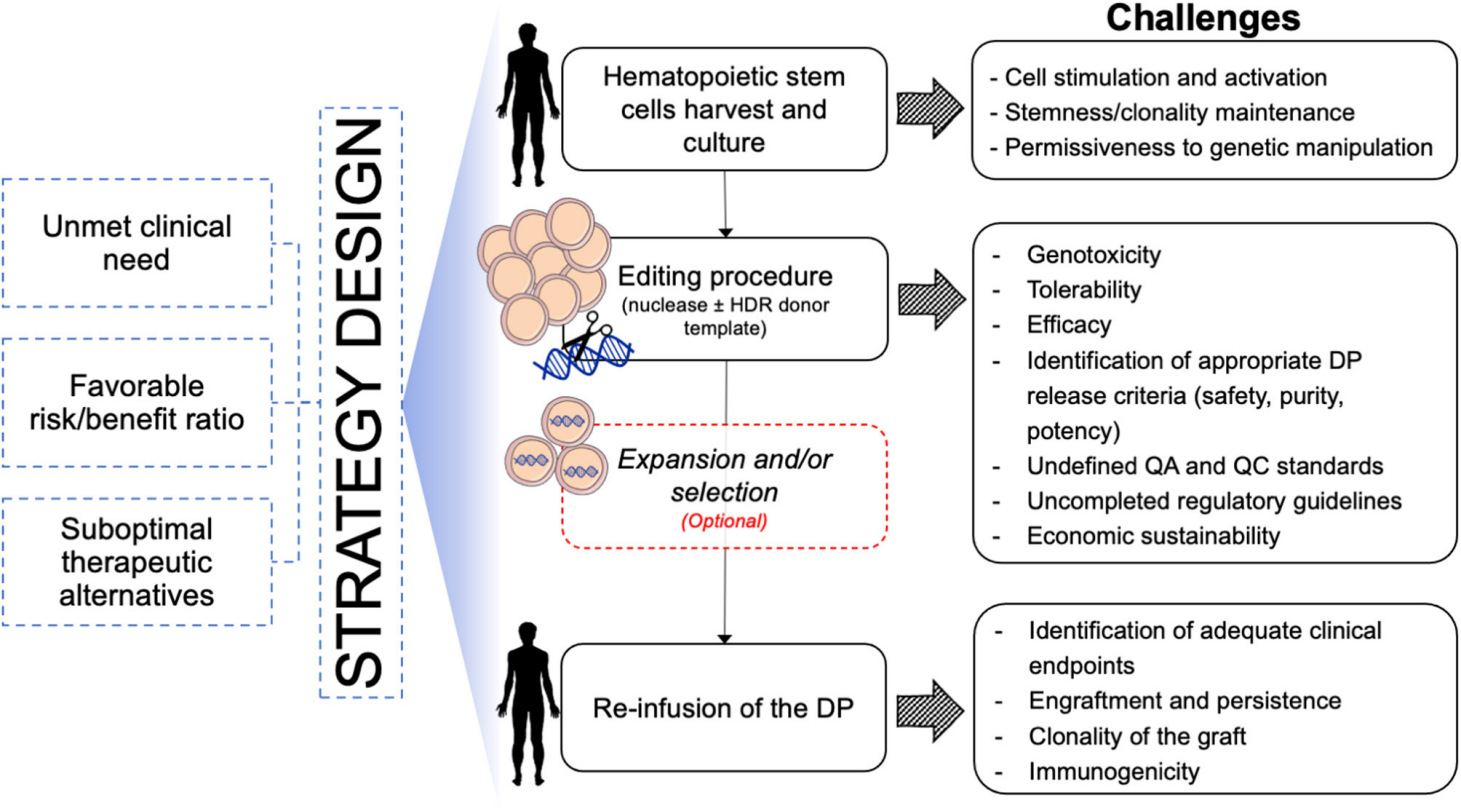
Schematic of challenges toward clinical translation of HSPC gene editing for hematological diseases. DP, Drug Product; QA, Quality Assay; QC, Quality Control.

In the last decades, the gene and cell therapy field faced an exponential expansion thanks to the accumulation of several clinical successes of both *ex vivo* and *in vivo* gene transfer approaches. The results of these studies are also providing important information about cell manufacturing, vector development and therapeutic efficacy, which represent a solid background and increase the expectation for the development of more precise gene editing approaches. Despite several similarities between the manufacturing of gene transferred and gene edited HSPCs (e.g., conditioning regimens for HSPC collection and transplantation, protocols for *in vitro* CD34+ cell selection, activation, culture, and transduction), the editing procedure requires additional and peculiar manipulation steps that only now will encounter their first clinical validation (Figure 3). Among these, the electroporation process used for the delivery of the editing components and the transduction of HSPCs with AAV6 vectors represent the innovations linked to major unknowns. In fact, there is still no direct evidence that these procedures will actually allow long-term engraftment of edited HSCs in a human subject. While state-of-the-art xenotransplantation studies on immunodeficient mice are showing promising results, critical species-specific differences in the procedure for the gene editing of murine and non-human primates (NHP) HSPCs significantly affect the yield, fitness, and potential immunogenicity of the cellular product, thus limiting the predictive value of such pre-clinical models (Schiroli et al., 2017; Kim et al., 2018; Humbert et al., 2019; Wilkinson et al., 2021). Moreover, the scaling-up of lab-grade to clinical-grade processes requires implementation of adequate manufacturing facility that support scalability of the procedures while encompassing the complex requirements to meet current good manufacturing practices (cGMP). The ideal cell manufacturing process needs to be robust, reproducible and cost-effective to be extended to multiple therapeutic applications. Finally, the safety, purity, and potency for the end-of-process cellular products need to be carefully defined to meet quality-control standards and regulatory agencies guidelines, which however still need to be tailored, based on accumulation of additional scientific knowledge. Indeed, manufacturing of edited HSPCs is still at the very beginning of clinical testing and regulatory agencies have to closely collaborate with scientists to identify the critical requirements that would better fit the needs of these advanced therapy medicinal products (ATMPs). Some clinical studies implying gene disruption in HSPCs with CRISPR-Cas9 (Frangoul et al., 2021) (NCT03745287/NCT03655678) are currently revealing precious information to prepare this transition. As light is shed on the effective and potential safety issues, it will become easier to define the appropriate framework of safety standards for subsequent applications.

### Tracking Edited HSPCs to Assess Safety, Efficacy, and Persistence of the Therapeutic Gene Edited Product

The study design of phase I/II HSPC gene editing clinical trials cannot exempt by the identification of a panel of adequate safety and efficacy endpoints, which would allow investigators to assess whether the proposed treatment meet the therapeutic expectations with a favorable risk/benefit ratio. However, clinical readouts must be complemented by *ad hoc* molecular analyses aimed at assessing long-term engraftment, persistence and multilineage differentiation potential of edited HSPCs, e.g., by the quantification of the editing efficiency at the on-target locus over time and across hematopoietic cell lineages during patients' follow up. Moreover, the assessment of genomic integrity as well as nuclease activity at validated off-target sites (if any) both in the manufactured cell product and within the patients' graft would comprehensively characterize the engineered cell product and early identify clonal drifts driven by the expansion of hematopoietic clones harboring structural genomic abnormalities or unintended editing outcomes.

In this framework, monitoring the clonal composition of the edited cell graft would provide precious additional information about the efficiency of the manufacturing process, the long-term multilineage repopulating potential of human edited HSPCs and the safety profile of the therapeutic approach. Quantification of the indel diversity within gene-edited alleles can function as a surrogate readout of clonal complexity of the edited cell population to track the dynamics of edited clones (McKenna et al., 2016; Kalhor et al., 2018; Román-Rodríguez et al., 2019; Ferrari et al., 2020). Recent studies in NHP models have shown a remarkable reduction of clonal complexity from the infused cell product to the graft with a direct correlation between the number of multilineage repopulating clones and the infused dose of edited cells (Demirci et al., 2020). Still, the preferential generation of specific edits by NHEJ repair (van Overbeek et al., 2016) and the occurrence of biallelic modifications might not provide sufficient complexity of the gene-edited allele population to exhaustively investigate clonal composition. The use of unique molecular identifier (e.g., random DNA sequences used as surrogate barcodes) embedded in the HDR template would enable tracking of HDR-edited clones, which would be otherwise indistinguishable from each other due to the high-fidelity nature of the HDR. HSPC editing with barcoded templates have uncovered the oligoclonal composition of the human HDR-edited xenograft in immunodeficient mice (Ferrari et al., 2020; Sharma et al., 2021) mainly attributable to the biological impact of the editing procedure, although most of the engrafting clones still retained multilineage and self-renewal potential. While the generation and the use of barcoded HDR template libraries with suitable complexity for clinical application would be extremely challenging and may rise theoretical safety concerns due to the random generation and integration of potentially functional/regulatory DNA sequences (e.g., transcription factor binding sites), this tool may be useful to validate the manufacturing process and the use of improved editing protocols.

Overall, preclinical observations of clonal dynamics reassure about long-term persistence of edited HSPCs after transplantation, even though the aforementioned loss of clonal complexity from the infused product to the edited cell graft prompts implementation and optimization of low-burden manufacturing processes, as well as monitoring clonal composition of the graft in first-in-man HSPC gene editing clinical trials.

### Economic Sustainability of ATMPs Based on Edited HSPCs

HSPC gene editing is a form of personalized medicine currently entailing complex and costly procedures, especially regarding the manufacturing and delivery processes, which are rising prices of such ATMPs to millions of US dollars. As occurred with gene replacement therapies, some of which have already reached Food and Drug Administration (FDA) and European Medicines Agency (EMA) approval for commercialization (Touchot and Flume, 2017), such high costs might be sustainable for the development of better treatments for ultra-rare and rare disorders, particularly in some countries. Indeed, (i) a single administration of the therapy may establish stable benefits with a substantial saving on the cost of repeated life-long administration of conventional therapies and (ii) the limited numbers of treated patients will not significantly impact the refunding system, except for the likely need to split the payment of a one-time treatment and distribute the credit along multiple years, thus mimicking the burden of a life-long therapy. Yet, now that the bar of these advanced therapeutic approaches has been elevated to reach more frequent diseases affecting a large number of people, such as hemoglobinopathies, this business model might result to be inadequate. The risk is that such high costs for ATMP production might impose socio-economic limits that could impair access to these new therapies and/or constrain their sustainable commercialization, thus ultimately affecting their availability for the patients (Wilson and Carroll, 2019). Nevertheless, the rapidly expanding technological advances in the gene therapy field are broadly considered a bottomless source of solutions for the aforementioned problems. The development of more efficient vector production systems, such advanced packaging cell lines or improved purification strategies (Grieger et al., 2016; Kotin and Snyder, 2017) as well as the implementation of small, automated, closed systems for cell manufacturing, which enable the de-centralized “point-of-care” generation of cellular therapies, will further ease the clinical testing of gene edited ATMPs and will soon significantly reduce their manufacturing costs. Indeed, the potential for an economically sustainable marketing of *ex vivo* gene therapies is well-supported by the exploding interest of big pharma's and venture capitals in the field, which foresee a favorable financial balance for these advanced therapies in the near future.

## Gene Addition vs. Gene Editing: Overlapping or Divergent Potential?

Gene transfer by RVs constituted a milestone in the history of gene therapy. Unfortunately, early enthusiasm following the therapeutic potential for congenital immunodeficiencies was suddenly quenched by the frequent malignant transformation of transduced cells (Cavazzana-Calvo et al., 2000; Hacein-Bey-Abina et al., 2003a,b, 2008; Howe et al., 2008; Stein et al., 2010; Braun et al., 2014), due to the overexpression of proto-oncogenes triggered by enhancer sequences within the U3 region of the 5′ long terminal repeat (LTR) of the nearby integrated vector. The transition from the RV to the LV platform and the generation of self-inactivating vectors by deletion of the LTR enhancer sequences resulted in a safer integration profile (Montini et al., 2006) and led to widespread use of modern LV for a number of diseases, such as hemoglobinopathies, enzymopathies and congenital immunodeficiencies (Sessa et al., 2016; Fraldi et al., 2018; Thompson et al., 2018; Mamcarz et al., 2019; Marktel et al., 2019; Kohn et al., 2020).

Insofar as variability in inter-cellular vector copy number, semi-random integration pattern, and gross regulation of cassette expression are not a priori an issue, LVs are arguably the best available platform for gene addition. However, some genotoxicity events might still occur upon LV integration, such as the vector-mediated disruption of relevant genomic elements or tumor suppressor genes and/or the generation of aberrant splicing variants of endogenous genes fused to the transgene cassette (Cavazzana-Calvo et al., 2010). Furthermore, the restoration of a complex physiological regulation of the transgene in the limiting size of the vector cassette may represent an additional problem in some disease contexts. The semi-random integration pattern of RVs and LVs discourages their discourages their use for those diseases where unregulated expression of the corrective gene might have potentially dangerous consequences, such as the case of genes that have a direct impact on cell proliferation and/or differentiation or genes that need a high expression level to restore their physiologic function. These disease categories include several primary immunodeficiencies, such as Hyper IgM 1 syndrome (Brown et al., 1998; Sacco et al., 2000; Hubbard et al., 2016; Kuo et al., 2018; Vavassori et al., 2021), RAG1/RAG2 deficiency (Villa et al., 2020) and, possibly, Fanconi anemia (Román-Rodríguez et al., 2019; van de Vrugt et al., 2019) for which the presumed increased risk of cell transformation or inadequate expression currently limit the development of competitive gene addition approaches and that can thus represent a suitable setting for a first in human testing of site-specific gene editing approaches. Moreover, therapeutic genome editing also extends the possible applications of precise DNA surgery to several vacant clinical contexts that cannot be efficiently addressed by conventional gene therapy strategies. Among others, the generation of HIV-resistant CCR5 knock-out cells (Xu et al., 2017, 2019), the deletion of regulatory regions (Bauer et al., 2013) or the correction of dominant negative mutations (Nasri et al., 2020) are applications that require exquisitely site-specific action of programmable enzymes.

Beyond the aforementioned examples, the potential applications of gene addition and gene editing overlap to a significant extent. Indeed, some degree of competition may arise for specific diseases whereby on-site restoration of gene function is expected to confer benefits in terms of expression. For instance, LV-based gene therapy for WAS reduced bleeding events but did not fully restore platelet levels (Ferrua et al., 2019). While the culprit is not entirely clear (Fischer, 2019), it has been postulated that full reconstitution of *in situ* physiological expression is required to fully correct the phenotype, thus opening the door to gene editing strategies (Rai et al., 2020). Another paradigmatic case is that of hemoglobinopathies, whereby clinical benefit may theoretically be achieved by (i) *HBB* gene addition/correction, or (ii) restoration of Hb-F expression by inactivating *BCL11A* (Sankaran et al., 2008; Basak et al., 2015). On one hand, *HBB* gene addition has proven to be feasible for both SCD and β-thalassemia (Thompson et al., 2018; Marktel et al., 2019), apparently leaving less room for HDR-based *HBB* editing (Dever et al., 2016; DeWitt et al., 2016; Kuo et al., 2018; Park et al., 2019; Pattabhi et al., 2019). On the other hand, suppression of *BCL11A* has been successfully achieved both with a LV encoding for a *BCL11A*-specific short hairpin RNA (Esrick et al., 2021), as well as by CRISPR-mediated knockout (Frangoul et al., 2021). Safety, efficacy and market success of these novel therapies, irrespectively of the platform, are anyhow expected to be benchmarked against betibeglogene autotemcel (Zynteglo), which has recently been approved by the FDA for the treatment of β-thalassemia (while the approval for SCD is still pending). As hemoglobinopathies are relatively frequent, it is possible that both gene addition and gene editing therapies with the same indication will be granted market approval, both in the USA and in other countries. This will allow for real-life side-by-side comparison of the different technologies in a not-so-distant future, which will highlight their respective advantages and disadvantages despite likely leaving space for more than a single winner, as normally occurs with other more conventional therapies (Fernandes et al., 2020).

Overall, the current efficiency of HDR-mediated gene correction is significantly lower than that achievable with LVs, thus diminishing its competitive advantage for a number of applications whereby high fraction of corrected cells is required. Still, the sword of Damocles of genotoxicity is hanging on both LV and gene editing platforms, either due to integration and inactivation of cancer suppressor genes, or to genomic rearrangements and off-target effects.

## Conclusions

The outstanding advantages and the current technological limitations of targeted genome editing are the main weights in the two sides of the scale when considering the opportunity of translating intriguing new therapeutic approaches into clinics. However, their “weight” might remarkably change depending on the target disease. Therefore, the decision to move gene editing toward human testing requires a case-by-case assessment and must be balanced against clinical need. In this scenario, the presence of competing treatments, either as standard of care or under clinical evaluation, and the costs of developing and commercializing ATMPs, might further restrict the space for the application of HSPC gene editing in blood disorders (Wilson and Carroll, 2019).

Ultimately, the rationale of testing novel gene editing-based strategies depends on the presumed benefit offered to the patient with respect to his prognosis with the best available therapy. It is reasonable to offer HSPC gene editing based products at first to patients with no alternative options and a dismal prognosis or to those for who the standard of care is presumed to be more toxic, such as those affected by severe congenital immunodeficiencies or DNA repair defects. Clinical testing of gene editing approaches in these applications would provide a first detailed characterization of their safety profile. This would also allow to define the appropriate assays to follow the dynamics of unwanted genomic events in time and establish their clinical relevance, setting the thresholds to manage the genotoxic risk. These data would then pave the way for their application to other diseases with a less dismal prognosis and alternative therapies, such as HIV and enzymopathies.

## Author Contributions

SF, VV, and DC conceived and wrote the manuscript. AJac, MC, and AJav contributed with ideas and discussion and wrote the manuscript. PG organized, supervised, and wrote the manuscript. All authors contributed to the article and approved the submitted version.

## Conflict of Interest

SF, VV, AJac, MC, and PG are inventors of patent applications on gene editing in HSPCs and cell selection owned and managed by the San Raffaele Scientific Institute and the Telethon Foundation. The remaining authors declare that the research was conducted in the absence of any commercial or financial relationships that could be construed as a potential conflict of interest.

## References

[B1] Abraham A. Hsieh M. Eapen M. Fitzhugh C. Carreras J. Keesler D.. (2017). Relationship between mixed donor–recipient chimerism and disease recurrence after hematopoietic cell transplantation for sickle cell disease. Biol. Blood Marrow Transplant. 23, 2178–2183. 10.1016/j.bbmt.2017.08.038 28882446 PMC5782809

[B2] Agudelo D. Duringer A. Bozoyan L. Huard C. C. Carter S. Loehr J.. (2017). Marker-free coselection for CRISPR-driven genome editing in human cells. Nat. Methods 14, 615–620. 10.1038/nmeth.4265 28417998

[B3] Aiuti A. Biasco L. Scaramuzza S. Ferrua F. Cicalese M. P. Baricordi C.. (2013). Lentiviral hematopoietic stem cell gene therapy in patients with wiskott-aldrich syndrome. Science 341:1233151. 10.1126/science.1233151 23845947 PMC4375961

[B4] Bak R. O. Dever D. P. Porteus M. H. (2018). CRISPR/Cas9 genome editing in human hematopoietic stem cells. Nat. Protoc. 13, 358–376. 10.1038/nprot.2017.143 29370156 PMC5826598

[B5] Barrangou R. Fremaux C. Deveau H. Richards M. Boyaval P. Moineau S.. (2007). CRISPR provides acquired resistance against viruses in prokaryotes. Science 315, 1709–1712. 10.1126/science.1138140 17379808

[B6] Basak A. Hancarova M. Ulirsch J. C. Balci T. B. Trkova M. Pelisek M.. (2015). BCL11A deletions result in fetal hemoglobin persistence and neurodevelopmental alterations. J. Clin. Invest. 125, 2363–2368. 10.1172/JCI81163 25938782 PMC4497765

[B7] Bauer D. E. Kamran S. C. Lessard S. Xu J. Fujiwara Y. Lin C.. (2013). An erythroid enhancer of BCL11A subject to genetic variation determines fetal hemoglobin level. Science 342, 253–257. 10.1126/science.1242088 24115442 PMC4018826

[B8] Beerman I. Seita J. Inlay M. A. Weissman I. L. Rossi D. J. (2014). Quiescent hematopoietic stem cells accumulate DNA damage during aging that is repaired upon entry into cell cycle. Cell Stem Cell 15, 37–50. 10.1016/j.stem.2014.04.016 24813857 PMC4082747

[B9] Bernardo M. E. Aiuti A. (2016). The role of conditioning in hematopoietic stem-cell gene therapy. Hum. Gene Ther. 27, 741–748. 10.1089/hum.2016.103 27530055

[B10] Biechonski S. Olender L. Zipin-Roitman A. Yassin M. Aqaqe N. Marcu-Malina V.. (2018). Attenuated DNA damage responses and increased apoptosis characterize human hematopoietic stem cells exposed to irradiation. Sci. Rep. 8:6071. 10.1038/s41598-018-24440-w 29666389 PMC5904119

[B11] Bogdanove A. J. Voytas D. F. (2011). TAL effectors: customizable proteins for DNA targeting. Science 333, 1843–1846. 10.1126/science.1204094 21960622

[B12] Boitano A. E. Wang J. Romeo R. Bouchez L. C. Parker A. E. Sutton S. E.. (2010). Aryl hydrocarbon receptor antagonists promote the expansion of human hematopoietic stem cells. Science 329, 1345–1348. 10.1126/science.1191536 20688981 PMC3033342

[B13] Bondar T. Medzhitov R. (2010). p53-Mediated hematopoietic stem and progenitor cell competition. Cell Stem Cell 6, 309–322. 10.1016/j.stem.2010.03.002 20362536 PMC2872065

[B14] Branzei D. Foiani M. (2008). Regulation of DNA repair throughout the cell cycle. Nat. Rev. Mol. Cell Biol. 9, 297–308. 10.1038/nrm2351 18285803

[B15] Braun C. J. Boztug K. Paruzynski A. Witzel M. Schwarzer A. Rothe M.. (2014). Gene therapy for Wiskott-Aldrich syndrome-long-term efficacy and genotoxicity. Sci. Transl. Med. 6:227ra33. 10.1126/scitranslmed.3007280 24622513

[B16] Brouns S. J. J. Jore M. M. Lundgren M. Westra E. R. Slijkhuis R. J. H. Snijders A. P. L.. (2008). Small CRISPR RNAs guide antiviral defense in prokaryotes. Science 321, 960–964. 10.1126/science.1159689 18703739 PMC5898235

[B17] Brown M. P. Topham D. J. Sangster M. Y. Zhao J. Flynn K. J. Surman S. L.. (1998). Thymic lymphoproliferative disease after successful correction of CD40 ligand deficiency by gene transfer in mice. Nat. Med. 4, 1253–1260. 10.1038/3233 9809548

[B18] Carroll D. (2014). Genome engineering with targetable nucleases. Annu. Rev. Biochem. 83, 409–439. 10.1146/annurev-biochem-060713-035418 24606144

[B19] Casini A. Olivieri M. Petris G. Montagna C. Reginato G. Maule G.. (2018). A highly specific SpCas9 variant is identified by *in vivo* screening in yeast. Nat. Biotechnol. 36, 265–271. 10.1038/nbt.4066 29431739 PMC6066108

[B20] Cavazzana-Calvo M. Hacein-Bey S. De Saint Basile G. Gross F. Yvon E. Nusbaum P.. (2000). Gene therapy of human severe combined immunodeficiency (SCID)-X1 disease. Science 288, 669–672. 10.1126/science.288.5466.669 10784449

[B21] Cavazzana-Calvo M. Payen E. Negre O. Wang G. Hehir K. Fusil F.. (2010). Transfusion independence and HMGA2 activation after gene therapy of human β-thalassaemia. Nature 467, 318–322. 10.1038/nature09328 20844535 PMC3355472

[B22] Ceccaldi R. Briot D. Larghero J. Vasquez N. D'Enghien C. D. Chamousset D.. (2011). Spontaneous abrogation of the G2 DNA damage checkpoint has clinical benefits but promotes leukemogenesis in Fanconi anemia patients. J. Clin. Invest. 121, 184–194. 10.1172/JCI43836 21183791 PMC3007150

[B23] Chapman J. R. Taylor M. R. G. Boulton S. J. (2012). Playing the end game: DNA double-strand break repair pathway choice. Mol. Cell 47, 497–510. 10.1016/j.molcel.2012.07.029 22920291

[B24] Charlesworth C. T. Camarena J. Cromer M. K. Vaidyanathan S. Bak R. O. Carte J. M.. (2018). Priming human repopulating hematopoietic stem and progenitor cells for Cas9/sgRNA gene targeting. Mol. Ther. Nucleic Acids 12, 89–104. 10.1016/j.omtn.2018.04.017 30195800 PMC6023838

[B25] Charpentier M. Khedher A. H. Y. Menoret S. Brion A. Lamribet K. Dardillac E.. (2018). CtIP fusion to Cas9 enhances transgene integration by homology-dependent repair. Nat. Commun. 9:1133. 10.1038/s41467-018-03475-7 29556040 PMC5859065

[B26] Cho S. W. Kim S. Kim Y. Kweon J. Kim H. S. Bae S.. (2014). Analysis of off-target effects of CRISPR/Cas-derived RNA-guided endonucleases and nickases. Genome Res. 24, 132–141. 10.1101/gr.162339.113 24253446 PMC3875854

[B27] Chu V. T. Weber T. Wefers B. Wurst W. Sander S. Rajewsky K.. (2015). Increasing the efficiency of homology-directed repair for CRISPR-Cas9-induced precise gene editing in mammalian cells. Nat. Biotechnol. 33, 543–548. 10.1038/nbt.3198 25803306

[B28] Cromer M. K. Vaidyanathan S. Ryan D. E. Curry B. Lucas A. B. Camarena J.. (2018). Global transcriptional response to CRISPR/Cas9-AAV6-based genome editing in CD34+ hematopoietic stem and progenitor cells. Mol. Ther. 26, 2431–2442. 10.1016/j.ymthe.2018.06.002 30005866 PMC6171165

[B29] Davis K. M. Pattanayak V. Thompson D. B. Zuris J. A. Liu D. R. (2015). Small molecule-triggered Cas9 protein with improved genome-editing specificity. Nat. Chem. Biol. 11, 316–318. 10.1038/nchembio.1793 25848930 PMC4402137

[B30] De Ravin S. S. Brault J. Meis R. J. Liu S. Li L. Pavel-Dinu M.. (2021). Enhanced homology-directed repair for highly efficient gene editing in hematopoietic stem/progenitor cells. Blood. 10.1182/blood.2020008503 33623984 PMC8120141

[B31] De Ravin S. S. Li L. Wu X. Choi U. Allen C. Koontz S.. (2017). CRISPR-Cas9 gene repair of hematopoietic stem cells from patients with X-linked chronic granulomatous disease. Sci. Transl. Med. 9:eaah3480. 10.1126/scitranslmed.aah3480 28077679

[B32] De Ravin S. S. Reik A. Liu P. Q. Li L. Wu X. Su L.. (2016). Targeted gene addition in human CD34 + hematopoietic cells for correction of X-linked chronic granulomatous disease. Nat. Biotechnol. 34, 424–429. 10.1038/nbt.3513 26950749 PMC4824656

[B33] Demirci S. Zeng J. Wu Y. Uchida N. Shen A. H. Pellin D.. (2020). BCL11A enhancer–edited hematopoietic stem cells persist in rhesus monkeys without toxicity. J. Clin. Invest. 130, 6677–6687. 10.1172/JCI140189 32897878 PMC7685754

[B34] Dever D. P. Bak R. O. Reinisch A. Camarena J. Washington G. Nicolas C. E.. (2016). CRISPR/Cas9 β-globin gene targeting in human haematopoietic stem cells. Nature 539, 384–389. 10.1038/nature20134 27820943 PMC5898607

[B35] DeWitt M. A. Magis W. Bray N. L. Wang T. Berman J. R. Urbinati F.. (2016). Selection-free genome editing of the sickle mutation in human adult hematopoietic stem/progenitor cells. Sci. Transl. Med. 8:360ra134. 10.1126/scitranslmed.aaf9336 27733558 PMC5500303

[B36] Di Micco R. Fumagalli M. Cicalese A. Piccinin S. Gasparini P. Luise C.. (2006). Oncogene-induced senescence is a DNA damage response triggered by DNA hyper-replication. Nature 444, 638–642. 10.1038/nature05327 17136094

[B37] Doudna J. A. (2020). The promise and challenge of therapeutic genome editing. Nature 578, 229–236. 10.1038/s41586-020-1978-5 32051598 PMC8992613

[B38] Eichler F. Duncan C. Musolino P. L. Orchard P. J. De Oliveira S. Thrasher A. J.. (2017). Hematopoietic stem-cell gene therapy for cerebral adrenoleukodystrophy. N. Engl. J. Med. 377, 1630–1638. 10.1056/NEJMoa1700554 28976817 PMC5708849

[B39] Enache O. M. Rendo V. Abdusamad M. Lam D. Davison D. Pal S.. (2020). Cas9 activates the p53 pathway and selects for p53-inactivating mutations. Nat. Genet. 52, 662–668. 10.1038/s41588-020-0623-4 32424350 PMC7343612

[B40] Esrick E. B. Lehmann L. E. Biffi A. Achebe M. Brendel C. Ciuculescu M. F.. (2021). Post-Transcriptional genetic silencing of BCL11A to treat sickle cell disease. N. Engl. J. Med. 384, 205–215. 10.1056/NEJMoa2029392 33283990 PMC7962145

[B41] Essers M. A. G. Offner S. Blanco-Bose W. E. Waibler Z. Kalinke U. Duchosal M. A.. (2009). IFNα activates dormant haematopoietic stem cells *in vivo*. Nature 458, 904–908. 10.1038/nature07815 19212321

[B42] Eyquem J. Mansilla-Soto J. Giavridis T. Van Der Stegen S. J. C. Hamieh M. Cunanan K. M.. (2017). Targeting a CAR to the TRAC locus with CRISPR/Cas9 enhances tumour rejection. Nature 543, 113–117. 10.1038/nature21405 28225754 PMC5558614

[B43] Fares I. Chagraoui J. Gareau Y. Gingras S. Ruel R. Mayotte N.. (2014). Pyrimidoindole derivatives are agonists of human hematopoietic stem cell self-renewal. Science 345, 1509–1512. 10.1126/science.1256337 25237102 PMC4372335

[B44] Fernandes J. F. Nichele S. Arcuri L. J. Ribeiro L. Zamperlini-Netto G. Loth G.. (2020). Outcomes after haploidentical stem cell transplantation with post-transplantation cyclophosphamide in patients with primary immunodeficiency diseases. Biol. Blood Marrow Transplant. 26, 1923–1929. 10.1016/j.bbmt.2020.07.003 32653621

[B45] Ferrari S. Jacob A. Beretta S. Unali G. Albano L. Vavassori V.. (2020). Efficient gene editing of human long-term hematopoietic stem cells validated by clonal tracking. Nat. Biotechnol. 38, 1298–1308. 10.1038/s41587-020-0551-y 32601433 PMC7610558

[B46] Ferrua F. Cicalese M. P. Galimberti S. Giannelli S. Dionisio F. Barzaghi F.. (2019). Lentiviral haemopoietic stem/progenitor cell gene therapy for treatment of Wiskott-Aldrich syndrome: interim results of a non-randomised, open-label, phase 1/2 clinical study. Lancet Haematol. 6, e239–e253. 10.1016/S2352-3026(19)30021-3 30981783 PMC6494976

[B47] Fischer A. (2019). Platelets are the achilles' heel of Wiskott-Aldrich syndrome. J. Allergy Clin. Immunol. 144, 668–670. 10.1016/j.jaci.2019.06.039 31310756

[B48] Fraldi A. Serafini M. Sorrentino N. C. Gentner B. Aiuti A. Bernardo M. E. (2018). Gene therapy for mucopolysaccharidoses: *in vivo* and *ex vivo* approaches. Ital. J. Pediatr. 44:130. 10.1186/s13052-018-0565-y 30442177 PMC6238250

[B49] Frangoul H. Altshuler D. Cappellini M. D. Chen Y.-S. Domm J. Eustace B. K.. (2021). CRISPR-Cas9 gene editing for sickle cell disease and β-thalassemia. N. Engl. J. Med. 384, 252–260. 10.1056/NEJMoa2031054 33283989

[B50] Fu Y. Sander J. D. Reyon D. Cascio V. M. Joung J. K. (2014). Improving CRISPR-Cas nuclease specificity using truncated guide RNAs. Nat. Biotechnol. 32, 279–284. 10.1038/nbt.2808 24463574 PMC3988262

[B51] Gabriel R. Lombardo A. Arens A. Miller J. C. Genovese P. Kaeppel C.. (2011). An unbiased genome-wide analysis of zinc-finger nuclease specificity. Nat. Biotechnol. 29, 816–823. 10.1038/nbt.1948 21822255

[B52] Gaj T. Gersbach C. A. Barbas C. F. (2013). ZFN, TALEN, and CRISPR/Cas-based methods for genome engineering. Trends Biotechnol. 31, 397–405. 10.1016/j.tibtech.2013.04.004 23664777 PMC3694601

[B53] Garaycoechea J. I. Crossan G. P. Langevin F. Mulderrig L. Louzada S. Yang F.. (2018). Alcohol and endogenous aldehydes damage chromosomes and mutate stem cells. Nature 553, 171–177. 10.1038/nature25154 29323295 PMC6047743

[B54] Garneau J. E. Dupuis M. È. Villion M. Romero D. A. Barrangou R. Boyaval P.. (2010). The CRISPR/cas bacterial immune system cleaves bacteriophage and plasmid DNA. Nature 468, 67–71. 10.1038/nature09523 21048762

[B55] Gaspar H. B. Cooray S. Gilmour K. C. Parsley K. L. Zhang F. Adams S.. (2011). Immunodeficiency: Hematopoietic stem cell gene therapy for adenosine deaminase-deficient severe combined immunodeficiency leads to long-term immunological recovery and metabolic correction. Sci. Transl. Med. 3:97ra80. 10.1126/scitranslmed.3002716 21865538

[B56] Genovese P. Schiroli G. Escobar G. Di Tomaso T. Firrito C. Calabria A.. (2014). Targeted genome editing in human repopulating haematopoietic stem cells. Nature 510, 235–240. 10.1038/nature13420 24870228 PMC4082311

[B57] Ghosh S. Thrasher A. J. Gaspar H. B. (2015). Gene therapy for monogenic disorders of the bone marrow. Br. J. Haematol. 171, 155–170. 10.1111/bjh.13520 26044877

[B58] Greaves M. (2018). A causal mechanism for childhood acute lymphoblastic leukaemia. Nat. Rev. Cancer 18, 471–484. 10.1038/s41568-018-0015-6 29784935 PMC6986894

[B59] Grieger J. C. Soltys S. M. Samulski R. J. (2016). Production of recombinant adeno-associated virus vectors using suspension HEK293 cells and continuous harvest of vector from the culture media for GMP FIX and FLT1 clinical vector. Mol. Ther. 24, 287–297. 10.1038/mt.2015.187 26437810 PMC4817810

[B60] Gutschner T. Haemmerle M. Genovese G. Draetta G. F. Chin L. (2016). Post-translational regulation of Cas9 during G1 enhances homology-directed repair. Cell Rep. 14, 1555–1566. 10.1016/j.celrep.2016.01.019 26854237

[B61] Haapaniemi E. Botla S. Persson J. Schmierer B. Taipale J. (2018). CRISPR-Cas9 genome editing induces a p53-mediated DNA damage response. Nat. Med. 24, 927–930. 10.1038/s41591-018-0049-z 29892067

[B62] Hacein-Bey-Abina S. Garrigue A. Wang G. P. Soulier J. Lim A. Morillon E.. (2008). Insertional oncogenesis in 4 patients after retrovirus-mediated gene therapy of SCID-X1. J. Clin. Invest. 118, 3132–3142. 10.1172/JCI35700 18688285 PMC2496963

[B63] Hacein-Bey-Abina S. von Kalle C. Schmidt M. Le Deist F. Wulffraat N. McIntyre E.. (2003a). A serious adverse event after successful gene therapy for X-linked severe combined immunodeficiency. N. Engl. J. Med. 348, 255–256. 10.1056/NEJM200301163480314 12529469

[B64] Hacein-Bey-Abina S. Von Kalle C. Schmidt M. McCormack M. P. Wulffraat N. Leboulch P.. (2003b). LMO2-associated clonal T cell proliferation in two patients after gene therapy for SCID-X1. Science 302, 415–419. 10.1126/science.1088547 14564000

[B65] Haeussler M. Schönig K. Eckert H. Eschstruth A. Mianné J. Renaud J. B.. (2016). Evaluation of off-target and on-target scoring algorithms and integration into the guide RNA selection tool CRISPOR. Genome Biol. 17:148. 10.1186/s13059-016-1012-2 27380939 PMC4934014

[B66] Handel E.-M. Cathomen T. (2011). Zinc-finger nuclease based genome surgery: its all about specificity. Curr. Gene Ther. 11, 28–37. 10.2174/156652311794520120 21182467

[B67] Hanlon K. S. Kleinstiver B. P. Garcia S. P. Zaborowski M. P. Volak A. Spirig S. E.. (2019). High levels of AAV vector integration into CRISPR-induced DNA breaks. Nat. Commun. 10:4439. 10.1038/s41467-019-12449-2 31570731 PMC6769011

[B68] Hendel A. Bak R. O. Clark J. T. Kennedy A. B. Ryan D. E. Roy S.. (2015). Chemically modified guide RNAs enhance CRISPR-Cas genome editing in human primary cells. Nat. Biotechnol. 33, 985–989. 10.1038/nbt.3290 26121415 PMC4729442

[B69] Heyer W. D. Ehmsen K. T. Liu J. (2010). Regulation of homologous recombination in eukaryotes. Annu. Rev. Genet. 44, 113–139. 10.1146/annurev-genet-051710-150955 20690856 PMC4114321

[B70] Hirsch M. L. (2015). Adeno-associated virus inverted terminal repeats stimulate gene editing. Gene Ther. 22, 190–195. 10.1038/gt.2014.109 25503695 PMC4388141

[B71] Hoggatt J. Singh P. Sampath J. Pelus L. M. (2009). Prostaglandin E2 enhances hematopoietic stem cell homing, survival, and proliferation. Blood 113, 5444–5455. 10.1182/blood-2009-01-201335 19324903 PMC2689046

[B72] Howe S. J. Mansour M. R. Schwarzwaelder K. Bartholomae C. Hubank M. Kempski H.. (2008). Insertional mutagenesis combined with acquired somatic mutations causes leukemogenesis following gene therapy of SCID-X1 patients. J. Clin. Invest. 118, 3143–3150. 10.1172/JCI35798 18688286 PMC2496964

[B73] Hsu P. D. Scott D. A. Weinstein J. A. Ran F. A. Konermann S. Agarwala V.. (2013). DNA targeting specificity of RNA-guided Cas9 nucleases. Nat. Biotechnol. 31, 827–832. 10.1038/nbt.2647 23873081 PMC3969858

[B74] Hu J. H. Miller S. M. Geurts M. H. Tang W. Chen L. Sun N.. (2018). Evolved Cas9 variants with broad PAM compatibility and high DNA specificity. Nature 556, 57–63. 10.1038/nature26155 29512652 PMC5951633

[B75] Hubbard B. P. Badran A. H. Zuris J. A. Guilinger J. P. Davis K. M. Chen L.. (2015). Continuous directed evolution of DNA-binding proteins to improve TALEN specificity. Nat. Methods 12, 939–942. 10.1038/nmeth.3515 26258293 PMC4589463

[B76] Hubbard N. Hagin D. Sommer K. Song Y. Khan I. Clough C.. (2016). Targeted gene editing restores regulated CD40L function in X-linked hyper-IgM syndrome. Blood 127, 2513–2522. 10.1182/blood-2015-11-683235 26903548

[B77] Humbert O. Radtke S. Samuelson C. Carrillo R. R. Perez A. M. Reddy S. S.. (2019). Therapeutically relevant engraftment of a CRISPR-Cas9-edited HSC-enriched population with HbF reactivation in nonhuman primates. Sci. Transl. Med. 11:eaaw3768. 10.1126/scitranslmed.aaw3768 31366580 PMC8407476

[B78] Ihry R. J. Worringer K. A. Salick M. R. Frias E. Ho D. Theriault K.. (2018). p53 inhibits CRISPR-Cas9 engineering in human pluripotent stem cells. Nat. Med. 24, 939–946. 10.1038/s41591-018-0050-6 29892062

[B79] Isalan M. (2012). Zinc-finger nucleases: how to play two good hands. Nat. Methods 9, 32–34. 10.1038/nmeth.1805 22205514

[B80] Jayavaradhan R. Pillis D. M. Goodman M. Zhang F. Zhang Y. Andreassen P. R.. (2019). CRISPR-Cas9 fusion to dominant-negative 53BP1 enhances HDR and inhibits NHEJ specifically at Cas9 target sites. Nat. Commun. 10:2866. 10.1038/s41467-019-10735-7 31253785 PMC6598984

[B81] Jinek M. Chylinski K. Fonfara I. Hauer M. Doudna J. A. Charpentier E. (2012). A programmable dual-RNA-guided DNA endonuclease in adaptive bacterial immunity. Science 337, 816–821. 10.1126/science.1225829 22745249 PMC6286148

[B82] Kalhor R. Kalhor K. Mejia L. Leeper K. Graveline A. Mali P.. (2018). Developmental barcoding of whole mouse via homing CRISPR. Science 361:eaat9804. 10.1126/science.aat9804 30093604 PMC6139672

[B83] Kim D. Bae S. Park J. Kim E. Kim S. Yu H. R.. (2015). Digenome-seq: genome-wide profiling of CRISPR-Cas9 off-target effects in human cells. Nat. Methods 12, 237–243. 10.1038/nmeth.3284 25664545

[B84] Kim M. Y. Yu K. R. Kenderian S. S. Ruella M. Chen S. Shin T. H.. (2018). Genetic Inactivation of CD33 in hematopoietic stem cells to enable CAR T cell immunotherapy for acute myeloid leukemia. Cell 173, 1439–1453.e19. 10.1016/j.cell.2018.05.013 29856956 PMC6003425

[B85] Kim S. Kim D. Cho S. W. Kim J. Kim J. S. (2014). Highly efficient RNA-guided genome editing in human cells via delivery of purified Cas9 ribonucleoproteins. Genome Res. 24, 1012–1019. 10.1101/gr.171322.113 24696461 PMC4032847

[B86] Kleinstiver B. P. Pattanayak V. Prew M. S. Tsai S. Q. Nguyen N. T. Zheng Z.. (2016). High-fidelity CRISPR-Cas9 nucleases with no detectable genome-wide off-target effects. Nature 529, 490–495. 10.1038/nature16526 26735016 PMC4851738

[B87] Kleinstiver B. P. Prew M. S. Tsai S. Q. Topkar V. V. Nguyen N. T. Zheng Z.. (2015). Engineered CRISPR-Cas9 nucleases with altered PAM specificities. Nature 523, 481–485. 10.1038/nature14592 26098369 PMC4540238

[B88] Kohn D. B. Booth C. Kang E. M. Pai S. Y. Shaw K. L. Santilli G.. (2020). Lentiviral gene therapy for X-linked chronic granulomatous disease. Nat. Med. 26, 200–206. 10.1038/s41591-019-0735-5 31988463 PMC7115833

[B89] Kosicki M. Tomberg K. Bradley A. (2018). Repair of double-strand breaks induced by CRISPR–Cas9 leads to large deletions and complex rearrangements. Nat. Biotechnol. 36, 765–771. 10.1038/nbt.4192 30010673 PMC6390938

[B90] Kotin R. M. Snyder R. O. (2017). Manufacturing clinical grade recombinant adeno-associated virus using invertebrate cell lines. Hum. Gene Ther. 28, 350–360. 10.1089/hum.2017.042 28351174

[B91] Kuo C. Y. Long J. D. Campo-Fernandez B. de Oliveira S. Cooper A. R. Romero Z.. (2018). Site-Specific gene editing of human hematopoietic stem cells for X-linked hyper-IgM syndrome. Cell Rep. 23, 2606–2616. 10.1016/j.celrep.2018.04.103 29847792 PMC6181643

[B92] Labun K. Guo X. Chavez A. Church G. Gagnon J. A. Valen E. (2019). Accurate analysis of genuine CRISPR editing events with ampliCan. Genome Res. 29, 843–847. 10.1101/gr.244293.118 30850374 PMC6499316

[B93] Lee C. M. Cradick T. J. Bao G. (2016). The Neisseria meningitidis CRISPR-Cas9 system enables specific genome editing in mammalian cells. Mol. Ther. 24, 645–654. 10.1038/mt.2016.8 26782639 PMC4786937

[B94] Lee H. J. Kim E. Kim J. S. (2010). Targeted chromosomal deletions in human cells using zinc finger nucleases. Genome Res. 20, 81–89. 10.1101/gr.099747.109 19952142 PMC2798833

[B95] Lee J. K. Jeong E. Lee J. Jung M. Shin E. Kim Y.. (2018). Directed evolution of CRISPR-Cas9 to increase its specificity. Nat. Commun. 9:3048. 10.1038/s41467-018-05477-x 30082838 PMC6078992

[B96] Lim Y. Bak S. Y. Sung K. Jeong E. Lee S. H. Kim J. S.. (2016). Structural roles of guide RNAs in the nuclease activity of Cas9 endonuclease. Nat. Commun. 7:13350. 10.1038/ncomms13350 27804953 PMC5097132

[B97] Lipton J. M. Ellis S. R. (2009). Diamond-Blackfan anemia: diagnosis, treatment, and molecular pathogenesis. Hematol. Oncol. Clin. North Am. 23, 261–282. 10.1016/j.hoc.2009.01.004 19327583 PMC2886591

[B98] Liu J. Guo Y. M. Hirokawa M. Iwamoto K. Ubukawa K. Michishita Y.. (2012). A synthetic double-stranded RNA, poly I: C, induces a rapid apoptosis of human CD34+ cells. Exp. Hematol. 40, 330–341. 10.1016/j.exphem.2011.12.002 22198151

[B99] Lombardo A. Genovese P. Beausejour C. M. Colleoni S. Lee Y. L. Kim K. A.. (2007). Gene editing in human stem cells using zinc finger nucleases and integrase-defective lentiviral vector delivery. Nat. Biotechnol. 25, 1298–1306. 10.1038/nbt1353 17965707

[B100] Mamcarz E. Zhou S. Lockey T. Abdelsamed H. Cross S. J. Kang G.. (2019). Lentiviral gene therapy combined with low-dose busulfan in infants with SCID-X1. N. Engl. J. Med. 380, 1525–1534. 10.1056/NEJMoa1815408 30995372 PMC6636624

[B101] Marktel S. Scaramuzza S. Cicalese M. P. Giglio F. Galimberti S. Lidonnici M. R.. (2019). Intrabone hematopoietic stem cell gene therapy for adult and pediatric patients affected by transfusion-dependent ß-thalassemia. Nat. Med. 25, 234–241. 10.1038/s41591-018-0301-6 30664781

[B102] Maruyama T. Dougan S. K. Truttmann M. C. Bilate A. M. Ingram J. R. Ploegh H. L. (2015). Increasing the efficiency of precise genome editing with CRISPR-Cas9 by inhibition of nonhomologous end joining. Nat. Biotechnol. 33, 538–542. 10.1038/nbt.3190 25798939 PMC4618510

[B103] McKenna A. Findlay G. M. Gagnon J. A. Horwitz M. S. Schier A. F. Shendure J. (2016). Whole-organism lineage tracing by combinatorial and cumulative genome editing. Science 353:aaf7907. 10.1126/science.aaf7907 27229144 PMC4967023

[B104] Miller J. C. Holmes M. C. Wang J. Guschin D. Y. Lee Y. L. Rupniewski I.. (2007). An improved zinc-finger nuclease architecture for highly specific genome editing. Nat. Biotechnol. 25, 778–785. 10.1038/nbt1319 17603475

[B105] Moehle E. A. Rock J. M. Lee Y. L. Jouvenot Y. DeKelver R. C. Gregory P. D.. (2007). Targeted gene addition into a specified location in the human genome using designed zinc finger nucleases. Proc. Natl. Acad. Sci. U.S.A. 104, 3055–3060. 10.1073/pnas.0611478104 17360608 PMC1802009

[B106] Montini E. Cesana D. Schmidt M. Sanvito F. Ponzoni M. Bartholomae C.. (2006). Hematopoietic stem cell gene transfer in a tumor-prone mouse model uncovers low genotoxicity of lentiviral vector integration. Nat. Biotechnol. 24, 687–696. 10.1038/nbt1216 16732270

[B107] Mu W. Tang N. Cheng C. Sun W. Wei X. Wang H. (2019). *In vitro* transcribed sgRNA causes cell death by inducing interferon release. Protein Cell 10, 461–465. 10.1007/s13238-018-0605-9 30618028 PMC6538590

[B108] Müller M. Lee C. M. Gasiunas G. Davis T. H. Cradick T. J. Siksnys V.. (2016). Streptococcus thermophilus CRISPR-Cas9 systems enable specific editing of the human genome. Mol. Ther. 24, 636–644. 10.1038/mt.2015.218 26658966 PMC4786917

[B109] Naldini L. (2019). Genetic engineering of hematopoiesis: current stage of clinical translation and future perspectives. EMBO Mol. Med. 11:e9958. 10.15252/emmm.201809958 30670463 PMC6404113

[B110] Nasri M. Ritter M. Mir P. Dannenmann B. Aghaallaei N. Amend D.. (2020). CRISPR/Cas9-mediated ELANE knockout enables neutrophilic maturation of primary hematopoietic stem and progenitor cells and induced pluripotent stem cells of severe congenital neutropenia patients. Haematologica 105, 598–609. 10.3324/haematol.2019.221804 31248972 PMC7049355

[B111] Nelson C. E. Wu Y. Gemberling M. P. Oliver M. L. Waller M. A. Bohning J. D.. (2019). Long-term evaluation of AAV-CRISPR genome editing for duchenne muscular dystrophy. Nat. Med. 25, 427–432. 10.1038/s41591-019-0344-3 30778238 PMC6455975

[B112] Nihongaki Y. Kawano F. Nakajima T. Sato M. (2015). Photoactivatable CRISPR-Cas9 for optogenetic genome editing. Nat. Biotechnol. 33, 755–760. 10.1038/nbt.3245 26076431

[B113] Nishimasu H. Shi X. Ishiguro S. Gao L. Hirano S. Okazaki S.. (2018). Engineered CRISPR-Cas9 nuclease with expanded targeting space. Science 361, 1259–1262. 10.1126/science.aas9129 30166441 PMC6368452

[B114] Nozawa T. Furukawa N. Aikawa C. Watanabe T. Haobam B. Kurokawa K.. (2011). CRISPR inhibition of prophage acquisition in Streptococcus pyogenes. PLoS ONE 6:e19543. 10.1371/journal.pone.0019543 21573110 PMC3089615

[B115] Park S. H. Lee C. M. Dever D. P. Davis T. H. Camarena J. Srifa W.. (2019). Highly efficient editing of the β-globin gene in patient-derived hematopoietic stem and progenitor cells to treat sickle cell disease. Nucleic Acids Res. 47, 7955–7972. 10.1093/nar/gkz475 31147717 PMC6735704

[B116] Pattabhi S. Lotti S. N. Berger M. P. Singh S. Lux C. T. Jacoby K.. (2019). In Vivo Outcome of homology-directed repair at the HBB Gene in HSC using alternative donor template delivery methods. Mol. Ther. Nucleic Acids 17, 277–288. 10.1016/j.omtn.2019.05.025 31279229 PMC6611979

[B117] Pattanayak V. Lin S. Guilinger J. P. Ma E. Doudna J. A. Liu D. R. (2013). High-throughput profiling of off-target DNA cleavage reveals RNA-programmed Cas9 nuclease specificity. Nat. Biotechnol. 31, 839–843. 10.1038/nbt.2673 23934178 PMC3782611

[B118] Pavani G. Laurent M. Fabiano A. Cantelli E. Sakkal A. Corre G.. (2020). *Ex vivo* editing of human hematopoietic stem cells for erythroid expression of therapeutic proteins. Nat. Commun. 11:3778. 10.1038/s41467-020-17552-3 32728076 PMC7391635

[B119] Pavel-Dinu M. Wiebking V. Dejene B. T. Srifa W. Mantri S. Nicolas C. E.. (2019). Gene correction for SCID-X1 in long-term hematopoietic stem cells. Nat. Commun. 10, 1634. 10.1038/s41467-019-09614-y 30967552 PMC6456568

[B120] Petrillo C. Thorne L. G. Unali G. Schiroli G. Giordano A. M. S. Piras F.. (2018). Cyclosporine H overcomes innate immune restrictions to improve lentiviral transduction and gene editing in human hematopoietic stem cells. Cell Stem Cell 23, 820–832.e9. 10.1016/j.stem.2018.10.008 30416070 PMC6292841

[B121] Piras F. Kajaste-Rudnitski A. (2020). Antiviral immunity and nucleic acid sensing in haematopoietic stem cell gene engineering. Gene Ther. 28, 16–28. 10.1038/s41434-020-0175-3 32661282 PMC7357672

[B122] Piras F. Riba M. Petrillo C. Lazarevic D. Cuccovillo I. Bartolaccini S.. (2017). Lentiviral vectors escape innate sensing but trigger p53 in human hematopoietic stem and progenitor cells. EMBO Mol. Med. 9, 1198–1211. 10.15252/emmm.201707922 28667090 PMC5582409

[B123] Rai R. Romito M. Rivers E. Turchiano G. Blattner G. Vetharoy W.. (2020). Targeted gene correction of human hematopoietic stem cells for the treatment of Wiskott - Aldrich Syndrome. Nat. Commun. 11:4034. 10.1038/s41467-020-17626-2 32788576 PMC7423939

[B124] Ran F. A. Cong L. Yan W. X. Scott D. A. Gootenberg J. S. Kriz A. J.. (2015). *In vivo* genome editing using *Staphylococcus aureus* Cas9. Nature 520, 186–191. 10.1038/nature14299 25830891 PMC4393360

[B125] Richardson C. D. Kazane K. R. Feng S. J. Zelin E. Bray N. L. Schäfer A. J.. (2018). CRISPR–Cas9 genome editing in human cells occurs via the Fanconi anemia pathway. Nat. Genet. 50, 1132–1139. 10.1038/s41588-018-0174-0 30054595

[B126] Román-Rodríguez F. J. Ugalde L. Álvarez L. Díez B. Ramírez M. J. Risueño C.. (2019). NHEJ-Mediated repair of CRISPR-Cas9-induced DNA breaks efficiently corrects mutations in HSPCs from patients with fanconi anemia. Cell Stem Cell 25, 607–621.e7. 10.1016/j.stem.2019.08.016 31543367

[B127] Romero Z. Lomova A. Said S. Miggelbrink A. Kuo C. Y. Campo-Fernandez B.. (2019). Editing the sickle cell disease mutation in human hematopoietic stem cells: comparison of endonucleases and homologous donor templates. Mol. Ther. 27, 1389–1406. 10.1016/j.ymthe.2019.05.014 31178391 PMC6697408

[B128] Roth T. L. Puig-Saus C. Yu R. Shifrut E. Carnevale J. Li P. J.. (2018). Reprogramming human T cell function and specificity with non-viral genome targeting. Nature 559, 405–409. 10.1038/s41586-018-0326-5 29995861 PMC6239417

[B129] Sacco M. G. Ungari M. Catò E. M. Villa A. Strina D. Notarangelo L. D.. (2000). Lymphoid abnormalities in CD40 ligand transgenic mice suggest the need for tight regulation in gene therapy approaches to hyper immunoglobulin M (IgM) syndrome. Cancer Gene Ther. 7, 1299–1306. 10.1038/sj.cgt.7700232 11059686

[B130] Sadelain M. Papapetrou E. P. Bushman F. D. (2012). Safe harbours for the integration of new DNA in the human genome. Nat. Rev. Cancer 12, 51–58. 10.1038/nrc3179 22129804

[B131] Sankaran V. G. Menne T. F. Xu J. Akie T. E. Lettre G. Van Handel B.. (2008). Human fetal hemoglobin expression is regulated by the developmental stage-specific repressor BCL11A. Science 322, 1839–1842. 10.1126/science.1165409 19056937

[B132] Sato T. Onai N. Yoshihara H. Arai F. Suda T. Ohteki T. (2009). Interferon regulatory factor-2 protects quiescent hematopoietic stem cells from type I interferon-dependent exhaustion. Nat. Med. 15, 696–700. 10.1038/nm.1973 19483695

[B133] Schiroli G. Conti A. Ferrari S. della Volpe L. Jacob A. Albano L.. (2019). Precise gene editing preserves hematopoietic stem cell function following transient p53-mediated DNA damage response. Cell Stem Cell 24, 551–565.e8. 10.1016/j.stem.2019.02.019 30905619 PMC6458988

[B134] Schiroli G. Ferrari S. Conway A. Jacob A. Capo V. Albano L.. (2017). Preclinical modeling highlights the therapeutic potential of hematopoietic stem cell gene editing for correction of SCID-X1. Sci. Transl. Med. 9:eaan0820. 10.1126/scitranslmed.aan0820 29021165

[B135] Sessa M. Lorioli L. Fumagalli F. Acquati S. Redaelli D. Baldoli C.. (2016). Lentiviral haemopoietic stem-cell gene therapy in early-onset metachromatic leukodystrophy: an ad-hoc analysis of a non-randomised, open-label, phase 1/2 trial. Lancet 388, 476–487. 10.1016/S0140-6736(16)30374-9 27289174

[B136] Shapiro J. Iancu O. Jacobi A. M. McNeill M. S. Turk R. Rettig G. R.. (2020). Increasing CRISPR efficiency and measuring its specificity in HSPCs using a clinically relevant system. Mol. Ther. Methods Clin. Dev. 17, 1097–1107. 10.1016/j.omtm.2020.04.027 32478125 PMC7251314

[B137] Sharma R. Dever D. P. Lee C. M. Azizi A. Pan Y. Camarena J.. (2021). The TRACE-Seq method tracks recombination alleles and identifies clonal reconstitution dynamics of gene targeted human hematopoietic stem cells. Nat. Commun. 12:472. 10.1038/s41467-020-20792-y 33473139 PMC7817666

[B138] Shin J. J. Schröder M. S. Caiado F. Wyman S. K. Bray N. L. Bordi M.. (2020). Controlled cycling and quiescence enables efficient HDR in engraftment-enriched adult hematopoietic stem and progenitor cells. Cell Rep. 32:108093. 10.1016/j.celrep.2020.108093 32877675 PMC7487781

[B139] Slaymaker I. M. Gao L. Zetsche B. Scott D. A. Yan W. X. Zhang F. (2016). Rationally engineered Cas9 nucleases with improved specificity. Science 351, 84–88. 10.1126/science.aad5227 26628643 PMC4714946

[B140] Soldi M. Sergi Sergi L. Unali G. Kerzel T. Cuccovillo I. Capasso P.. (2020). Laboratory-Scale lentiviral vector production and purification for enhanced *ex vivo* and *in vivo* genetic engineering. Mol. Ther. Methods Clin. Dev. 19, 411–425. 10.1016/j.omtm.2020.10.009 33294490 PMC7683235

[B141] Stein S. Ott M. G. Schultze-Strasser S. Jauch A. Burwinkel B. Kinner A.. (2010). Genomic instability and myelodysplasia with monosomy 7 consequent to EVI1 activation after gene therapy for chronic granulomatous disease. Nat. Med. 16, 198–204. 10.1038/nm.2088 20098431

[B142] Sternberg S. H. Redding S. Jinek M. Greene E. C. Doudna J. A. (2014). DNA interrogation by the CRISPR RNA-guided endonuclease Cas9. Nature 507, 62–67. 10.1038/nature13011 24476820 PMC4106473

[B143] Sweeney C. L. Zou J. Choi U. Merling R. K. Liu A. Bodansky A.. (2017). Targeted Repair of CYBB in X-CGD iPSCs requires retention of intronic sequences for expression and functional correction. Mol. Ther. 25, 321–330. 10.1016/j.ymthe.2016.11.012 28153086 PMC5368476

[B144] Takahashi K. Wang F. Kantarjian H. Doss D. Khanna K. Thompson E.. (2017). Preleukaemic clonal haemopoiesis and risk of therapy-related myeloid neoplasms: a case-control study. Lancet Oncol. 18, 100–111. 10.1016/S1470-2045(16)30626-X 27923552 PMC5405697

[B145] Thompson A. A. Walters M. C. Kwiatkowski J. Rasko J. E. J. Ribeil J.-A. Hongeng S.. (2018). Gene therapy in patients with transfusion-dependent β-thalassemia. N. Engl. J. Med. 378, 1479–1493. 10.1056/NEJMoa1705342 29669226

[B146] Touchot N. Flume M. (2017). Early insights from commercialization of gene therapies in Europe. Genes 8:78. 10.3390/genes8020078 28218692 PMC5333067

[B147] Tsai S. Q. Nguyen N. T. Malagon-Lopez J. Topkar V. V. Aryee M. J. Joung J. K. (2017). CIRCLE-seq: a highly sensitive *in vitro* screen for genome-wide CRISPR-Cas9 nuclease off-targets. Nat. Methods 14, 607–614. 10.1038/nmeth.4278 28459458 PMC5924695

[B148] Tsai S. Q. Zheng Z. Nguyen N. T. Liebers M. Topkar V. V. Thapar V.. (2015). GUIDE-seq enables genome-wide profiling of off-target cleavage by CRISPR-Cas nucleases. Nat. Biotechnol. 33, 187–198. 10.1038/nbt.3117 25513782 PMC4320685

[B149] Urnov F. D. Miller J. C. Lee Y. L. Beausejour C. M. Rock J. M. Augustus S.. (2005). Highly efficient endogenous human gene correction using designed zinc-finger nucleases. Nature 435, 646–651. 10.1038/nature03556 15806097

[B150] Urnov F. D. Rebar E. J. Holmes M. C. Zhang H. S. Gregory P. D. (2010). Genome editing with engineered zinc finger nucleases. Nat. Rev. Genet. 11, 636–646. 10.1038/nrg2842 20717154

[B151] Vakulskas C. A. Dever D. P. Rettig G. R. Turk R. Jacobi A. M. Collingwood M. A.. (2018). A high-fidelity Cas9 mutant delivered as a ribonucleoprotein complex enables efficient gene editing in human hematopoietic stem and progenitor cells. Nat. Med. 24, 1216–1224. 10.1038/s41591-018-0137-0 30082871 PMC6107069

[B152] van de Vrugt H. J. Harmsen T. Riepsaame J. Alexantya G. van Mil S. E. de Vries Y.. (2019). Effective CRISPR/Cas9-mediated correction of a Fanconi anemia defect by error-prone end joining or templated repair. Sci. Rep. 9:768. 10.1038/s41598-018-36506-w 30683899 PMC6347620

[B153] van Overbeek M. Capurso D. Carter M. M. Thompson M. S. Frias E. Russ C.. (2016). DNA repair profiling reveals nonrandom outcomes at Cas9-mediated breaks. Mol. Cell 63, 633–646. 10.1016/j.molcel.2016.06.037 27499295

[B154] Vavassori V. Mercuri E. Marcovecchio G. E. Castiello M. C. Schiroli G. Albano L.. (2021). Modeling, optimization, and comparable efficacy of T cell and hematopoietic stem cell gene editing for treating hyper-IgM syndrome. EMBO Mol. Med. 13:e13545. 10.15252/emmm.202013545 33475257 PMC7933961

[B155] Villa A. Capo V. Castiello M. C. (2020). Innovative cell-based therapies and conditioning to cure RAG deficiency. Front. Immunol. 11:607926. 10.3389/fimmu.2020.607926 33329604 PMC7711106

[B156] Voit R. A. Hendel A. Pruett-Miller S. M. Porteus M. H. (2014). Nuclease-mediated gene editing by homologous recombination of the human globin locus. Nucleic Acids Res. 42, 1365–1378. 10.1093/nar/gkt947 24157834 PMC3902937

[B157] Walasek M. A. van Os R. de Haan G. (2012). Hematopoietic stem cell expansion: challenges and opportunities. Ann. N. Y. Acad. Sci. 1266, 138–150. 10.1111/j.1749-6632.2012.06549.x 22901265

[B158] Walton R. T. Christie K. A. Whittaker M. N. Kleinstiver B. P. (2020). Unconstrained genome targeting with near-PAMless engineered CRISPR-Cas9 variants. Science 368, 290–296. 10.1126/science.aba8853 32217751 PMC7297043

[B159] Wang C. X. Cannon P. M. (2016). The clinical applications of genome editing in HIV. Blood 127, 2546–2552. 10.1182/blood-2016-01-678144 27053530 PMC4882804

[B160] Wang J. Exline C. M. Declercq J. J. Llewellyn G. N. Hayward S. B. Li P. W. L.. (2015). Homology-driven genome editing in hematopoietic stem and progenitor cells using ZFN mRNA and AAV6 donors. Nat. Biotechnol. 33, 1256–1263. 10.1038/nbt.3408 26551060 PMC4842001

[B161] Wang Q. Chen S. Xiao Q. Liu Z. Liu S. Hou P.. (2017). Genome modification of CXCR4 by Staphylococcus aureus Cas9 renders cells resistance to HIV-1 infection. Retrovirology 14:51. 10.1186/s12977-017-0375-0 29141633 PMC5688617

[B162] Wilkinson A. C. Dever D. P. Baik R. Camarena J. Hsu I. Charlesworth C. T.. (2021). Cas9-AAV6 gene correction of beta-globin in autologous HSCs improves sickle cell disease erythropoiesis in mice. Nat. Commun. 12:686. 10.1038/s41467-021-20909-x 33514718 PMC7846836

[B163] Wilson R. C. Carroll D. (2019). The daunting economics of therapeutic genome editing. Cris. J. 2, 280–284. 10.1089/crispr.2019.0052 31599686

[B164] Xiao Q. Chen S. Wang Q. Liu Z. Liu S. Deng H.. (2019). CCR5 editing by *Staphylococcus aureus* Cas9 in human primary CD4+ T cells and hematopoietic stem/progenitor cells promotes HIV-1 resistance and CD4+ T cell enrichment in humanized mice. Retrovirology 16:15. 10.1186/s12977-019-0477-y 31186067 PMC6560749

[B165] Xu K. Ren C. Liu Z. Zhang T. Zhang T. Li D.. (2015). Efficient genome engineering in eukaryotes using Cas9 from *Streptococcus thermophilus*. Cell. Mol. Life Sci. 72, 383–399. 10.1007/s00018-014-1679-z 25038777 PMC11113816

[B166] Xu L. Wang J. Liu Y. Xie L. Su B. Mou D.. (2019). CRISPR-edited stem cells in a patient with HIV and acute lymphocytic leukemia. N. Engl. J. Med. 381, 1240–1247. 10.1056/NEJMoa1817426 31509667

[B167] Xu L. Yang H. Gao Y. Chen Z. Xie L. Liu Y.. (2017). CRISPR/Cas9-MEDIATED ccr5 ablation in human hematopoietic stem/progenitor cells confers HIV-1 resistance *in vivo*. Mol. Ther. 25, 1782–1789. 10.1016/j.ymthe.2017.04.027 28527722 PMC5542791

[B168] Yeh C. D. Richardson C. D. Corn J. E. (2019). Advances in genome editing through control of DNA repair pathways. Nat. Cell Biol. 21, 1468–1478. 10.1038/s41556-019-0425-z 31792376

[B169] Zetsche B. Gootenberg J. S. Abudayyeh O. O. Slaymaker I. M. Makarova K. S. Essletzbichler P.. (2015a). Cpf1 is a single RNA-guided endonuclease of a class 2 CRISPR-Cas system. Cell 163, 759–771. 10.1016/j.cell.2015.09.038 26422227 PMC4638220

[B170] Zetsche B. Volz S. E. Zhang F. (2015b). A split-Cas9 architecture for inducible genome editing and transcription modulation. Nat. Biotechnol. 33, 139–142. 10.1038/nbt.3149 25643054 PMC4503468

[B171] Zink F. Stacey S. N. Norddahl G. L. Frigge M. L. Magnusson O. T. Jonsdottir I.. (2017). Clonal hematopoiesis, with and without candidate driver mutations, is common in the elderly. Blood 130, 742–752. 10.1182/blood-2017-02-769869 28483762 PMC5553576

[B172] Zonari E. Desantis G. Petrillo C. Boccalatte F. E. Lidonnici M. R. Kajaste-Rudnitski A.. (2017). Efficient *ex vivo* engineering and expansion of highly purified human hematopoietic stem and progenitor cell populations for gene therapy. Stem Cell Rep. 8, 977–990. 10.1016/j.stemcr.2017.02.010 28330619 PMC5390102

